# A Simple Route to Strong Carbon‐13 NMR Signals Detectable for Several Minutes

**DOI:** 10.1002/chem.201702767

**Published:** 2017-07-19

**Authors:** Soumya S. Roy, Philip Norcott, Peter J. Rayner, Gary G. R. Green, Simon B. Duckett

**Affiliations:** ^1^ Department of Chemistry University of York Heslington, York YO10 5DD UK; ^2^ York Neuroimaging Centre The Biocentre, York Science Park Innovation Way, Heslington York YO10 5NY UK

**Keywords:** hyperpolarization, long-lived singlet states, NMR spectroscopy, *para*-hydrogen, structure elucidation

## Abstract

Nuclear magnetic resonance (NMR) and magnetic resonance imaging (MRI) suffer from low sensitivity and limited nuclear spin memory lifetimes. Although hyperpolarization techniques increase sensitivity, there is also a desire to increase relaxation times to expand the range of applications addressable by these methods. Here, we demonstrate a route to create hyperpolarized magnetization in ^13^C nuclear spin pairs that last much longer than normal lifetimes by storage in a singlet state. By combining molecular design and low‐field storage with *para*‐hydrogen derived hyperpolarization, we achieve more than three orders of signal amplification relative to equilibrium Zeeman polarization and an order of magnitude extension in state lifetime. These studies use a range of specifically synthesized pyridazine derivatives and dimethyl *p*‐tolyl phenyl pyridazine is the most successful, achieving a lifetime of about 190 s in low‐field, which leads to a ^13^C‐signal that is visible for 10 minutes.

Although carbon is one of the most abundant elements in nature, its NMR‐active form carbon‐13 is present at only about a 1.1 % level which, when coupled with its low magnetogyric ratio, results in low detectability. Consequently, ^13^C magnetic resonance imaging (MRI) produces a negligible response when compared to proton measurement in the body, which is facile due to high water content and high sensitivity. ^13^C detection does, however, benefit from potentially long relaxation times when compared to those of the proton.

A number of methods, commonly known as hyperpolarization, exist that can increase NMR sensitivity in nuclei such as ^13^C and are being used to overcome these issues.^1^, ^2^ These approaches artificially increase the associated spin population differences between the energy levels that are probed. For example, Golman et al. reported a *para*‐hydrogen (*p*‐H_2_) induced nuclear polarization (PHIP) study,^3^, ^4^ which achieved the rapid in vivo detection of a ^13^C‐MRI response in 2001.^5^ Two years later, they described the results of a similar study using dissolution dynamic nuclear polarization (DNP),^6^ in which a normally inaccessible response was seen in vivo. Bhattacharya et al. have since incorporated *p*‐H_2_ into sodium 1–^13^C acetylene dicarboxylate to facilitate the collection of an arterial ^13^C‐MRI image of a rat brain.^7^ More recently, a DNP‐derived ^13^C‐MRI response with chemical shift resolution has been shown to distinguish different metabolic flux between normal and tumor cells in humans.^8^, ^9^, ^10^, ^11^ These studies illustrate the potential benefits to human health if such methods were to become widely accessible and hence establish the need for a rapid and low‐cost delivery method for long‐lived ^13^C hyperpolarization.

In this article, we demonstrate that the goal of rapidly producing a long‐lived ^13^C hyperpolarized response can be met by applying the signal amplification by reversible exchange (SABRE) effect.^12^, ^13^, ^14^ In SABRE, a catalyst reversibly binds *p*‐H_2_ and the substrate to transfer dormant spin order from *p*‐H_2_ into the substrate through the scalar‐coupling framework, as shown in Scheme 1. We use this approach here to hyperpolarize a series of coupled ^13^C spin‐pairs in a range of pyridazine derivatives, a motif that exhibits pharmacological activity.^15^, ^16^ Polarization is then stored in specially created singlet spin order to enable a response to be seen several minutes later. Although a range of nicotinamide‐ and pyridazine‐based substrates have been shown to deliver long‐lived ^1^H hyperpolarization,^17^, ^18^ and analogous ^15^N‐based singlets have been created by Warren and co‐workers,^19^ we believe the ^13^C responses reported here are significant due to the growing use of ^13^C‐MRI for in vivo study.

**Scheme 1:**
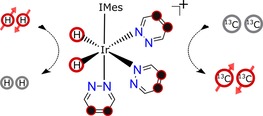
Schematic depiction of the SABRE hyperpolarization technique. IMes=1,3‐bis(2,4,6‐trimethylphenyl)imidazol‐2‐ylidene.

The term singlet (|*S*_0_⟩=(|*αβ*⟩−|*βα*⟩)√2) that is used here represents the spin‐zero magnetic alignment of a coupled spin‐1/2 system, the conversion of which into the associated triplet states (|*T*_0_⟩=(|*αβ*⟩+|*βα*⟩)√2; |*T*_1_⟩=|*αα*⟩; |*T*_−1_⟩=|*ββ*⟩) is symmetry‐forbidden. Consequently, any population difference that can be created between these singlet and triplet forms is expected to relax more slowly than the usual time constant *T*_1_.^20^ The symmetry properties that make such states long‐lived also make them challenging to generate and probe.^20^, ^21^ Levitt and co‐workers have demonstrated a number of strategies to do this in a range of chemically inequivalent spin systems^22^, ^23^, ^24^, ^25^, ^26^ and have achieved a lifetime of over one hour in an optimized chemical system at low field.^27^ However, when a substantial chemical shift difference exists between these spin‐pairs, the application of a spin‐lock, or sample‐shuttling to low field, is necessary to extend state lifetime.^22^, ^28^, ^29^ This effect has recently been illustrated by monitoring the effect of solvent‐dependent chemical‐shift changes.^17^, ^18^ Warren and co‐workers have reported a parallel approach that exploits magnetic inequivalence to create related singlet states.^21^, ^30^, ^31^, ^32^, ^33^ Thus, whereas SABRE has been shown to create hyperpolarized ^1^H‐ and ^15^N‐derived singlets, there is a need to expand these methods to ^13^C given the success of DNP.^8^, ^9^, ^10^, ^11^ However, ^13^C‐SABRE itself has currently seen limited application^34^ and reported efficiency gains are relatively low. We have now developed a molecular design strategy for use with SABRE and radio frequency (*rf*) excitation to achieve greater than 2 % net ^13^C polarization in a long‐lived form.

In this study, we employ magnetic and chemical inequivalence effects through the synthesis of specific substrates in which their carbon‐4 and carbon‐5 sites are ^13^C‐enriched, as detailed in Scheme 2 (full synthetic strategy and characterization data are available in the Supporting Information, Section S1–3). The *Type‐1* form agents exhibit chemically equivalent but magnetically inequivalent ^13^C spin‐pairs (▵*δ*=0) and have local *C*_2_ symmetry. The *Type‐2a* form is constructed such that R^1^ and R^2^ are chemically different and a small chemical‐shift difference results between the ^13^C spin‐pair (▵*δ*≠0). Chemical inequivalence is also derived by remote substitution at R^2^ and R^3^, in the *Type‐2b* agents of Scheme 2. Although these synthetic strategies allow access to two distinct classes of molecular system, our results illustrate that both are equally viable.

**Scheme 2:**
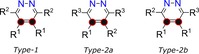
The molecular systems studied here are of *Type‐1*, which reflect a chemically equivalent but magnetically distinct ^13^C spin‐pair (black and red dots), or *Type‐2a* and *Type‐2b*, which reflect chemically inequivalent ^13^C spin‐pairs (R^1^≠R^2^≠R^3^).

To explore the singlet states of these systems, their NMR properties must first be analyzed. The *Type‐1* substrate, **1**, of Table 1 reflects an AA′XX′‐type spin system (Figure 1 a) and produces the ^13^C NMR spectrum shown in Figure 1 b. This trace illustrates the effect of magnetic inequivalence, but does not immediately yield the individual carbon–proton couplings (^2^ *J*_CH_ and ^3^ *J*_CH_) necessary to create a singlet state by the method of Warren,^21^ because the peak‐to‐peak separations reflect the mean value of the ^13^C‐^1^H *J*‐couplings (5.25 Hz=[^2^ *J*_CH_+^3^ *J*_CH_]/2). By employing a *J*‐synchronized experiment,^23^, ^30^, ^32^ it is possible to show that the difference in these *J*‐couplings is 3.1 Hz (see Section S5 in the Supporting Information). We harness this difference in coupling (Δ*J*_CH_) to populate the singlet state through *rf* pulse‐sequencing, as detailed in Figure 1 c. Table 1 details the chemical structures of *Type‐1* agents **1**–**3** that are examined here. A value of zero for Δ*J*_CH_ means that it is not possible to induce interconversion between the singlet and triplets forms through *rf* pulsing (e.g., agent **3**, see Section S5).^20^

**Table 1:** ^13^C (red/white dots) SABRE signal enhancement (*ϵ*) over the corresponding thermal measurement at 9.4 T after transfer at the indicated field (*G*), net polarization (*P*) and *T*_1_ and *T*_S_ lifetimes (s) of substrate **1**–**8** in high field (HF: 9.4 T) and low field (LF: ≈10 mT). The *J*‐coupling between the ^13^C spin‐pair was found to be about 58.5±2.0 Hz in all cases. The Δ*J*_CH_ values for *Type‐1* substrates, and the chemical shift difference (Δ*v*) for *Type‐2* substrates are noted.

Agent	Substrate structure	Enhancement (*ϵ*), transfer field, net polarization level *P* [%]	Lifetime [s]	Δ*J*_CH_* or Δ*v*@9.4 T [Hz]
**1**	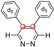	*ϵ*: 2500±300 @30 G *P*≈2.0	*T*_1_: 9.7±0.3 *T*_S(HF)_: 75±5.5 *T*_S(LF)_: 115±12	3.1±0.2*
**2**	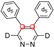	*ϵ*: 1600±280 @150 G *P*≈1.3	*T*_1_: 12.4±0.9 *T*_S(HF/LF)_: –	^2^ *J*_CD_≈0.4*
**3**	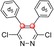	*ϵ*: 600±50 @20 mG *P*≈0.5	*T*_1_: 16.0±1.5 *T*_S(HF/LF)_: No access	0
**4**	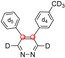	*ϵ*: 1600±300 @150 G *P*≈1.3	*T*_1_: 10.2±0.6 *T*_S(HF)_: 22±3.0 *T*_S(LF)_: 28±6.5	11.0±0.1
**5**	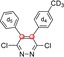	*ϵ*: 550±50 @5 mG *P*≈0.45	*T*_1_: 15.5±1.2 *T*_S(HF)_: 90±3.0 *T*_S(LF)_: 165±18	10.4±0.1
**6**	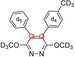	*ϵ*: 350±40 @10 mG *P*≈0.35	*T*_1_: 10.4±0.3 *T*_S(HF)_: 115±5.5 *T*_S(LF)_: 148±20	14.5±0.4
**7**	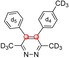	*ϵ*: 600±50 @1 mG *P*≈0.50	*T*_1_: 15.2±0.3 *T*_S(HF)_: 145±6.0 *T*_S(LF)_: 186±18	4.4±0.3
**8**	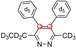	*ϵ*: 800±150 @10 mG *P*≈0.65	*T*_1_: 7.5±0.5 *T*_S(HF)_: <5 *T*_S(LF)_: 45±6.0	78.8±0.5

**Figure 1:**
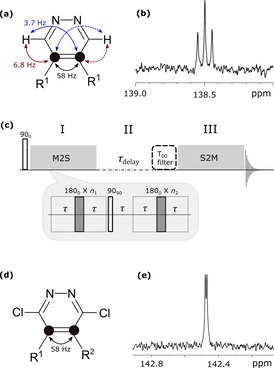
(a) Spin topology of the *Type‐1* agent **1**, showing the *J*‐couplings that exist between the ^1^H and ^13^C nuclei, in which R^1^=deuterated phenyl group; (b) corresponding ^13^C NMR spectrum of agent **1** in [D_4_]MeOH; (c) M2S‐S2M pulse sequence used here; (d) spin topology of *Type‐2* substrate **5** and corresponding ^13^C NMR spectrum in [D_4_]MeOH (e).

We also prepared agents **4**–**8**, which reflect a series of *Type‐2* molecular systems. Their spin system is illustrated in Figure 1 d, whereas Figure 1 e shows the ^13^C NMR spectrum of agent **5** in [D_4_]MeOH. In this case, the partially resolved 1.05 Hz (▵*δ* ^2^ *ν* ^2^/2*J*_CC_) splitting signifies that a strongly coupled ^13^C spin‐pair results when R^1^ and R^2^ are deuterated phenyl and *para*‐tolyl groups, respectively.

The pulse sequence that is used to create and examine the lifetime of the singlet state in these *Type‐1* and ‐*2* molecules consists of three parts, as detailed in Figure 1 c. Part **I** converts longitudinal magnetization into singlet order (M2S), part **II** preserves this singlet order, and part **III** converts it back into a visible form. The first and last steps are realized experimentally by a train of *n* 180° pulses that are separated by delay (*τ*), which is a molecule‐specific parameter. For the *Type‐1* system, **1** in which *J*_CC_≫*J*_HH_, Equations (1)–(3) provide *τ* and *n*.^30^, ^32^

(1) $$\tau =\frac{1}{\left(2\sqrt{\left(J{}_{\mathrm{CC}}+J{}_{\mathrm{HH}}\right){}^{2}+\left(\Delta J{}_{\mathrm{CH}}\right){}^{2}}\right)}$$

(2) $$n{}_{1}=\frac{\pi }{\left(2\tan{}^{-1}\left[\Delta J{}_{\mathrm{CH}}/\left(J{}_{\mathrm{CC}}+J{}_{\mathrm{HH}}\right)\right]\right)}$$

(3) $$n{}_{2}≃n{}_{1}/2$$

In contrast, in the case of the *Type‐2* spin systems (agents **4**–**8**), these parameters come from Equations (3)–(5) shown above and below.

(4) $$\tau =\frac{1}{\left(4\sqrt{J{}^{2}+\Delta \delta {}^{2}v{}^{2}}\right)}$$

(5) $$n{}_{1}=\frac{\pi }{\left(2\tan{}^{-1}\left[\Delta \delta \cdot v/J\right)]\right)}$$

Section S7 in the Supporting Information details these values for **1**–**8**. The resulting singlet states were then stored either in high field or in low field (after sample transfer). For **1**, the singlet state lifetimes (*T*_S_) were measured to be 75±5.5 and 115±12 s at high and low field, respectively. We therefore see about a 10‐fold increase over the 9.4 T *T*_1_ relaxation time of 9.7 s. The effect of a spin‐lock during high‐field storage proved to be minimal, increasing the *T*_S_ by only about 10 %. In the case of agent **5**, we achieved a *T*_S_ of 90±3 s in high field, which increases to 165±18 s in low‐field. Table 1 summarizes these values for agents **1**–**8** and confirms that this strategy allows the creation of long‐lived singlet states in these molecules. ^2^H‐labeled **7** contained the optimal molecular environment of the series, delivering a low‐field *T*_S_ of 186±18 s.

For **2**, the ^13^C‐^2^H couplings are too small to exploit the M2S sequence to prepare the singlet. For **4**, the singlet‐state lifetime proved low due to the ^13^C‐deuterium coupling, which provides a route to scalar relaxation.^35^ In **8**, the chemical shift difference between the ^13^C pairs is similar to the *J*‐coupling constant in high field and a low lifetime results but in low field this extends to 45 s. In contrast, agents **5**, **6** and **7** operate well in both low and high field, exhibiting lifetimes in excess of 150 s in low field.

A series of SABRE experiments were then undertaken to see if it was possible to create hyperpolarized longitudinal spin order within the ^13^C manifold of agents **1**–**8** (Table 1). This involved taking [D_4_]MeOH solutions that contained 20 mm of the substrate, and 5 mm of the IMes catalyst. *p*‐H_2_ gas was bubbled through the solution for 20 s in low field and the sample transferred into the NMR spectrometer for further analysis. Figure 2 highlights the results of this process, with the level of ^13^C polarization reaching about 2 % as compared to the corresponding thermal polarization of only 0.0008 % at 9.4 T in the case of agent **1** after relayed transfer from ^1^H–^13^C at 30 G (see Section S6 in the Supporting Information). No H/D‐exchange is observable on the timescale of the SABRE experiment. The relayed transfer process was then examined as a function of the magnetic field experienced by the sample, and three maxima were observed, at about 10 mG (using μ‐metal shield), 30 and 100 G. Simulation revealed the about 10 mG maxima is associated with direct hydride–carbon spin‐spin transfer by the ^4^ *J* ${}_{{}^{1}H\text{-}{}^{13}C}$ and ^5^ *J* ${}_{{}^{1}H\text{-}{}^{13}C}$ couplings in the catalyst. The remaining maxima appear to result from relayed transfer by the agents ^1^H response (see Section S4).

**Figure 2:**
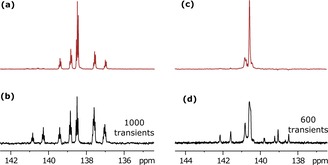
^13^C NMR spectra of **1** after (a) SABRE at a mixing field 5 mG and corresponding thermally equilibrated signal of 1000 transients. (c) Similar SABRE studies of **7** at a mixing field of 1 mG and (d) its thermal equilibrium spectra acquired by 600 transients.

When agent **2** is examined, the ^2^H labels should prevent the relayed response that is operating and restrict its transfer to the approximate 10 mG field range. Under these conditions, a strong ^13^C signal is seen. However, upon moving from 10–150 G, ^13^C and ^1^H SABRE enhanced signals are observed in the ^1^H and ^13^C frequency ranges. These results reveal readily detectable contributions from the ^2^H‐^1^H isotopologue, which is present at 1 %, through the observation of a ^13^C response that contains a *J* ${}_{{}^{1}H\text{-}{}^{13}C}$ splitting of 5.4 Hz. This reflects one of the challenges faced when working with hyperpolarization in so far as low‐concentration species can be readily detected. Agents **3** and **5**–**8** also require direct polarization transfer because there is no suitable relayed transfer pathway and they once again work well between 1 and 20 mG. These ^13^C hyperpolarization data are summarized in Table 1 (and Section S5). Polarization levels approaching 2 % are readily achieved, which would be expected to increase further through catalyst optimization.^36^ We then transferred the resulting ^13^C‐hyperpolarization into singlet order using the methods described earlier. The efficiency of singlet conversion in all successful cases was found to be in the range of 50–80 %.

Figure 3 shows the decay of the resulting hyperpolarized ^13^C singlet derived signals for agents **1**, **5**–**8** as a function of their storage time (*T*_S_) in low field. The ^13^C lifetimes proved to be directly comparable to those measured without hyperpolarization and signals can be readily observed for several minutes after creation when stored in a low‐field region. In the case of **7**, hyperpolarized signals were detectable for well over 10 mins.

**Figure 3:**
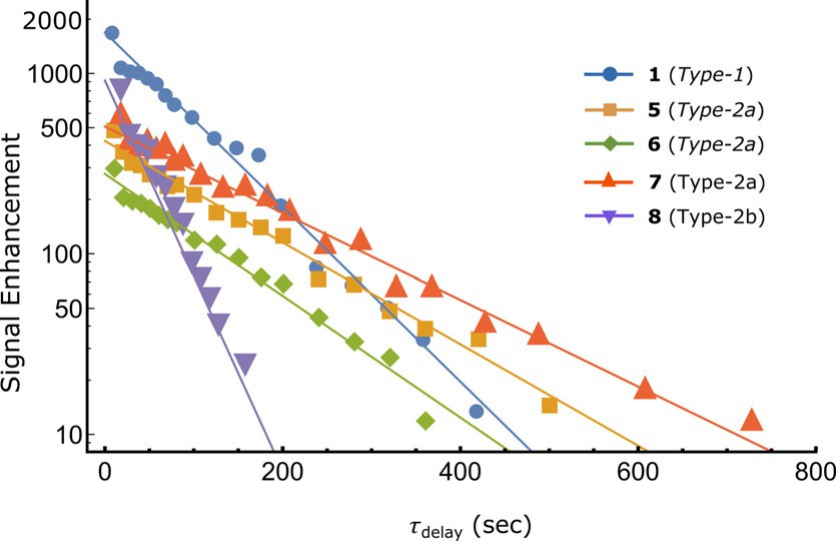
Hyperpolarized ^13^C singlet state decay (log10 scale) as a function of low‐field storage time (*τ*_delay_) for agents **1**, **5**–**8**. Results are summarized in Table 1.

In summary, we have demonstrated that a series of novel agents can be prepared that contain two adjacent ^13^C labels in addition to two nitrogen‐based lone pairs, which make them suitable for SABRE. Despite the weak *J*‐coupling that exists between the hydride ligands and the targeted ^13^C sites, we achieve a hyperpolarized response at the 2 % level. This hyperpolarization has then been efficiently converted into singlet spin order within the two ^13^C labels by *rf* excitation with a low‐field relaxation time of about 190 s being the result for deuterated dimethyl *p*‐tolyl phenyl pyridazine. This process has been exemplified for both magnetic and chemical inequivalence. Our method provides a fast and low‐cost technique to create ^13^C hyperpolarization in a reversible fashion with very little waste. Because of the simplicity of this approach, we envisage that this strategy will be adopted more widely to hyperpolarize related tracers. We are currently seeking to improve on the purity of these states to test the in vivo detection of these agents.

## Conflict of interest

The authors declare no conflict of interest.

## Supporting information

As a service to our authors and readers, this journal provides supporting information supplied by the authors. Such materials are peer reviewed and may be re‐organized for online delivery, but are not copy‐edited or typeset. Technical support issues arising from supporting information (other than missing files) should be addressed to the authors.

Supplementary
