# Supplementary material for: A Simple Route to Strong Carbon‐13 NMR Signals Detectable for Several Minutes
Source: Chemistry. 2017 Jul 19;23(44):10496–500. doi: 10.1002/chem.201702767 (PMC5582603; doi:10.1002/chem.201702767)
Supplement: Supplementary file 1 — Supplementary [file CHEM-23-10496-s001.pdf]

CHEMISTRY
A European Journal

Supporting Information

A Simple Route to Strong Carbon-13 NMR Signals Detectable for Several Minutes

Soumya S. Roy,[a] Philip Norcott,[a] Peter J. Rayner,[a] Gary G. R. Green,[b] and
Simon B. Duckett*[a]

chem_201702767_sm_miscellaneous_information.pdf

S1. Experimental procedures

S2. Catalyst

S3. Synthetic methods and characterization

S4. Theory and simulations

S5. Long-lived singlet states (LLS)

S6. NMR spectral data

S7. References

S1. Experimental procedure

All the experiments were performed in a 400 MHz Bruker Avance III series spectrometer equipped with a standard BBO broadband probe. We followed established approaches to use the SABRE hyperpolarization technique as described below. Hydrogen (H_2) enriched to 95% *para*-hydrogen (p-H_2) was employed in all the experiments related to SABRE and SABRE-LLS.

Shake & drop method: A simple approach to completing the initial SABRE experiments was followed: Solutions were prepared with appropriate ratios of substrate, catalyst, and solvent in a 5 mm NMR tube fitted with a J. Young's cap. The resulting solution was then degassed by three freeze-pump-thaw cycles which removes the majority of the dissolved oxygen in the solution. SABRE and SABRE-LLS experiments were performed by adding p-H_2 gas to the NMR tube and consequently mixing the solution at a specific magnetic field before quickly putting the NMR tube into the spectrometer for subsequent measurement. This method proves to be extremely effective and it takes less than a minute to complete a hyperpolarization experiment. See reference [1] for more details.

Flow method: This provides for a more systematic way of completing the experiments. The mixing chamber is placed inside a voltage-controlled copper-coiled electromagnetic field. The chamber itself is kept inside a double-layered mu-metal chamber to remove any stray magnetic field inside. A programmable microcontroller sets the desired parameters (e.g. p-H_2 bubbling time, mixing field, time to transfer etc.) to perform the whole experiment in an automatic fashion. The setup, shown in Figure S1, allow for repeated measurements and has a high level of reproducibility. More details about this procedure can be found in reference [2].

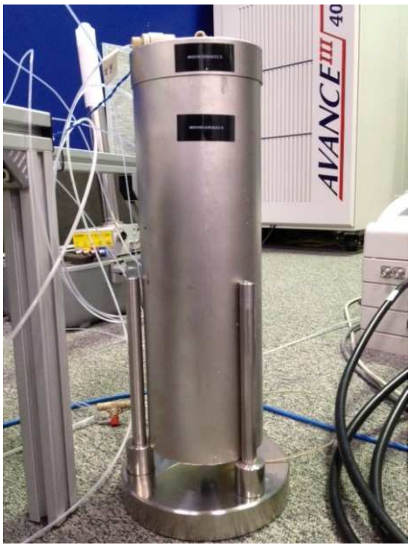

Figure S1: The automatic 'flow' mode polarizer used in this study.

Enhancement factor: The ^{13}C NMR SABRE enhancement level was calculated by applying the following formula

$$\varepsilon = \left(\frac{S_{HP}}{S_{Ref}}\right) * N_{Ref}$$

where S_{HP} denotes the hyperpolarized signal intensity, acquired in a single transient of 90° pulse. S_{Ref} represents the thermally polarized signal intensity added over N_{Ref} number of transients. Thermal signal were acquired overnight by a

specified number of 90° pulses with sufficient (at least 5 times T_1) relaxation delay between each scans. All NMR experimental parameters, except the number of scans, remain the same for hyperpolarized and thermal measurements.

Lifetime (T_1 and T_S) measurement:

We used a standard inversion recovery experiment to measure T_1 relaxation values under thermal conditions. Signal intensities were fitted to the equation: $M_z(\tau) = M_0(1 - 2e^{-\tau/T_1})$ while varying the delay times (τ).

Singlet lifetime (T_S) measurements were evaluated by varying the storage times (τ) in a series of M2S-S2M experiments.[3] Integrated signal intensities were fitted to the mono-exponential equation: $M_z(\tau) = M_0 e^{-\tau/T_S}$ to produce the T_S value. Here $M_z(\tau)$ represents the integrated signal amplitudes at time τ, while M_0 is a fitting constant.

Solvent: All the experiments were done in deuterated methanol unless otherwise stated.

Sample preparation: Samples were prepared with the following specifications:

(i) Sample prepared for 'shake & drop' method*:* 2 mg (3.12 μmol) of [IrCl(COD)(IMes)] precatalyst with a substrate loading that leads to a one-fold excess of added agent after formation of the active catalyst in 0.6 mL of solvent.

(ii) Sample prepared for 'flow' method*:* 10 mg (15.6 μmol) of [IrCl(COD)(IMes)] precatalyst with substrate loading that leads to a one-fold excess of agent after formation of the active catalyst in 3 mL of solvent.

S2. Catalyst

In this study, we used [IrCl(COD)(IMes)] as the catalyst precursor in all cases unless otherwise stated. It was synthesized in the laboratory following the standard literature procedure.[4] Here IMes stands for 1,3-bis(2,4,6-trimethyl-phenyl)imidazole-2-ylidene and COD = *cis,cis*-1,5-cyclooctadiene. A typical reaction of the catalyst with the substrate ligand (Sub) and *p*-H_2 is shown below. The substrate binds to the metal through a nitrogen center as described below.

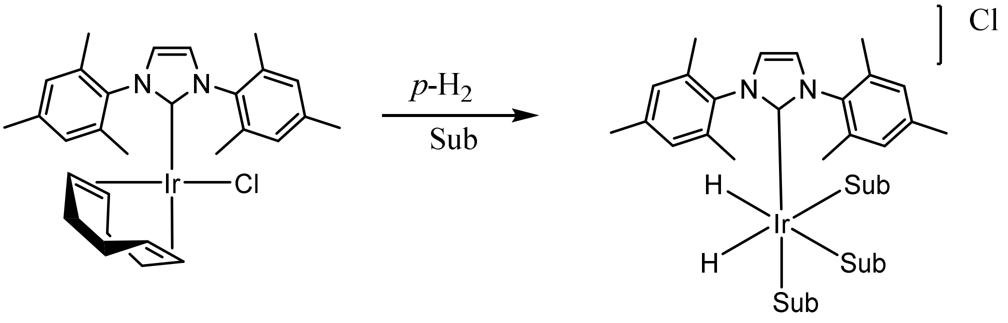

Figure S2: Schematic diagram showing the underlying reaction associated with SABRE.

In all cases reported in this study, the complex produces a single hydride resonance at ~δ –21.2 ± 0.4 ppm in the corresponding ^{1}H NMR spectrum as per the reaction shown in Figure S1. The process for pyridazine, and a series of its related forms have been extensively studied previously.[5-6]

S3. Synthetic Methods

S3.1 General

Distilled water was employed where detailed. Brine refers to a saturated aqueous solution of NaCl. THF was freshly distilled from sodium and benzophenone ketyl or dried using a Grubbs solvent purification system. All reactions were carried out under O_2-free N_2 unless otherwise stated.

Flash column chromatography was carried out using Fluka Chemie GmbH silica (220-440 mesh). Thin layer chromatography was carried out using Merck F_{254} aluminium-backed silica plates. ^{1}H (400 MHz) and ^{13}C (100.6 MHz) NMR spectra were recorded on a Bruker-400 instrument with an internal deuterium lock. Chemical shifts are quoted as parts per million and referenced to $CHCl_3$ (δ_H 7.27), $CDCl_3$ (δ_C 77.0). ^{13}C NMR spectra were recorded with broadband proton decoupling. ^{13}C NMR spectra were assigned using DEPT experiments when necessary. Coupling constants (*J*) are quoted in Hertz. Electrospray high and low resolution mass spectra were recorded on a Bruker Daltronics microOTOF spectrometer.

All commercial compounds listed were purchased from Sigma-Aldrich, Fluorochem or Alfa-Aesar and used as supplied unless otherwise stated. The following compounds were synthesised using literature procedures, di(d_5-phenyl)acetylene-1,2-$^{13}C_2$ (**S5**) and [IrCl(COD)(IMes)].[4]

S3.2 Synthetic Methods and Characterization

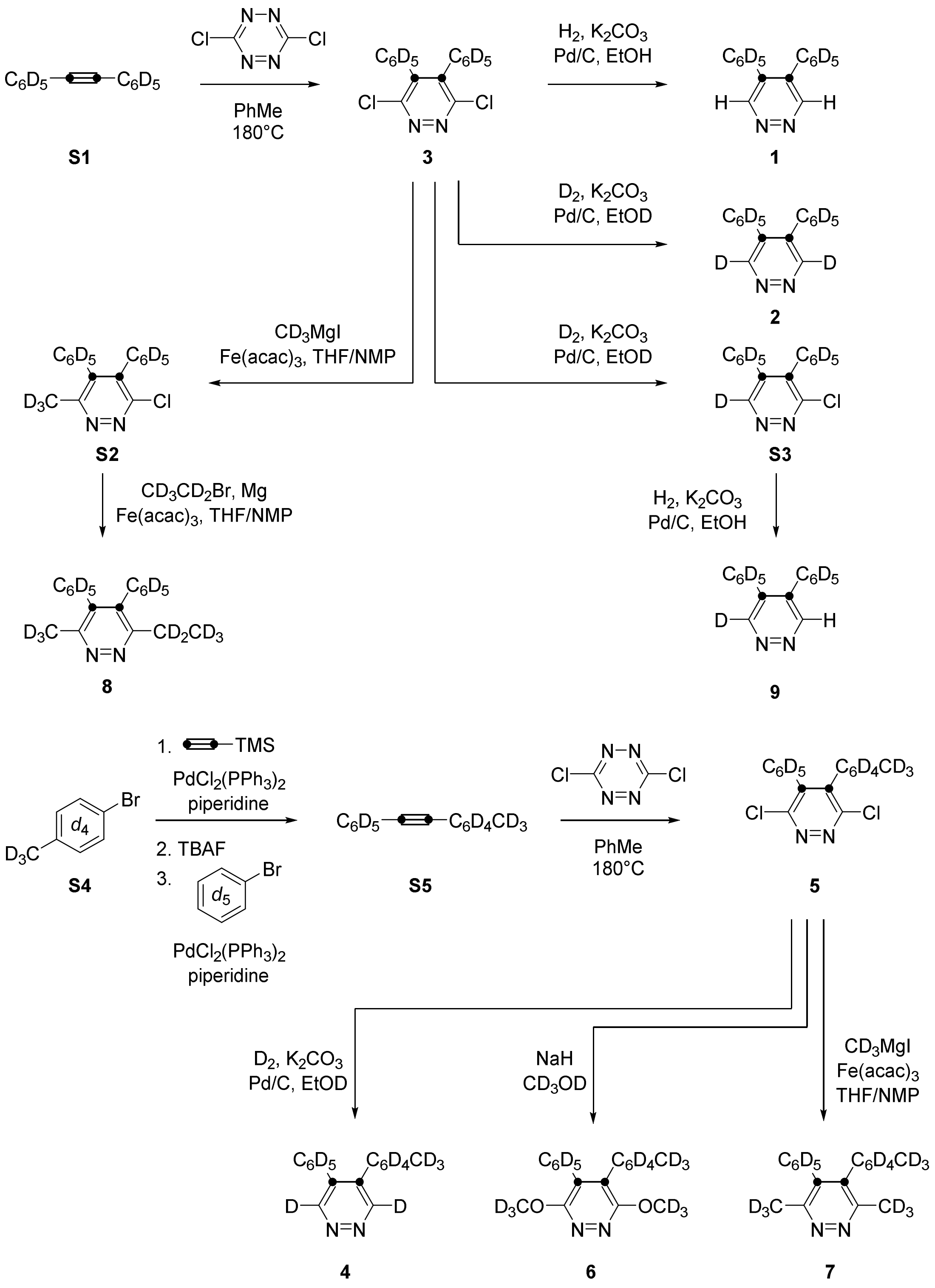

Scheme S1: Overview of the synthetic strategy to ^{13}C labelled substrates.

3,6-dichloro-4,5-di(d_5-phenyl)pyridazine-4,5-$^{13}C_2$ 3

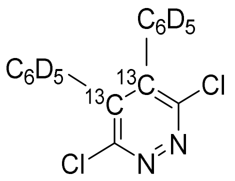

3

A solution of di(d_5-phenyl)acetylene-1,2-$^{13}C_2$ **S5** (265 mg, 1.64 mmol, 1.2 eq.) and 3,6-dichloro-1,2,4,5-tetrazine (260 mg, 1.37 mmol, 1 eq.) in PhMe (5 mL) was heated to 180°C for 6 hours under microwave irradiation. The mixture was concentrated under reduced pressure and purified by flash column chromatography with 1:9 EtOAc:hexane as eluent to give 3,6-dichloro-4-(d_7-*p*-tolyl)-5-(d_5-phenyl)pyridazine-4,5-$^{13}C_2$ **5** (392 mg, 86%) as a colorless solid, **^{13}C NMR** (101 MHz, $CDCl_3$) δ 141.7 (s); **MS** (ESI) *m/z* 313 [(M + H)$^+$, 100]; **HRMS** (ESI) *m/z* [M + H]$^+$ calculated for $C_{14}{}^{13}C_2Cl_2HD_{10}N_2^+$ 313.0989, found 313.0982 (+2.4 ppm error).

Comparison to 3,6-dichloro-4,5-di(d_5-phenyl)pyridazine (S6) with carbon at natural abundance

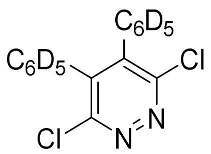

S6

A solution of di(d_5-phenyl)acetylene (145 mg, 0.96 mmol) and 3,6-dichloro-1,2,4,5-tetrazine (180 mg, 1.2 mmol, 1.2 eq.) in PhMe (4 mL) was heated to 180°C for 16 hours under microwave irradiation. The mixture was concentrated under reduced pressure and purified by flash column chromatography with 1:9 EtOAc:hexane as eluent to give 3,6-dichloro-4,5-di(d_5-phenyl)pyridazine **S6** (108 mg, 36%) as a light pink solid; **^{13}C NMR** (101 MHz, $CDCl_3$) δ 156.3 (s), 141.7 (s), 132.8 (s), 128.9 (t, J = 24.3 Hz), 128.6 (t, J = 24.6 Hz), 127.9 (t, J = 24.5 Hz); **MS** (ESI) *m/z* 311 [(M + H)$^+$, 100]; **HRMS** (ESI) *m/z* [M + H]$^+$ calculated for $C_{16}HD_{10}N_2Cl_2^+$ 311.0921, found 311.0918 (+3.2 ppm error).

4,5-di(d_5-phenyl)pyridazine-4,5-$^{13}C_2$ 1

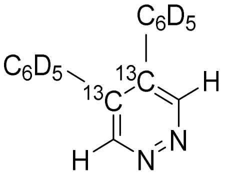

1

To a suspension of 3,6-dichloro-4,5-di(d_5-phenyl)pyridazine-4,5-$^{13}C_2$ (100 mg, 0.32 mmol), and K_2CO_3 (92 mg, 0.67 mmol, 2.1 eq.) in THF (10 mL) in a 30 mL Parr reactor was added 5% Pd/C (10 mg, 10 wt%). The reactor was sealed, purged with N_2, then pressurised with H_2 (8 bar). The reaction mixture was stirred at room temperature for 2 hours. The pressure was released and the suspension was filtered through Celite and washed with EtOAc. The filtrate was concentrated under reduced pressure and purified by flash column chromatography with EtOAc as eluent to give 4,5-di(d_5-phenyl)pyridazine-4,5-$^{13}C_2$ **1** (70 mg, 90%) as a white solid; **^{1}H NMR** (400 MHz, $CDCl_3$) δ 9.23 (t, J = 5.0 Hz, 2H); **^{13}C NMR** (101 MHz, $CDCl_3$) δ 137.2 (s); **MS** (ESI) *m/z* 245 [(M + H)$^+$, 100]; **HRMS** (ESI) *m/z* [M + H]$^+$ calculated for $C_{14}{}^{13}C_2H_3D_{10}N_2{}^+$ 245.1768, found 245.1768 (−0.1 ppm error).

Comparison to 4,5-di(d_5-phenyl)pyridazine (S7) with carbon at natural abundance

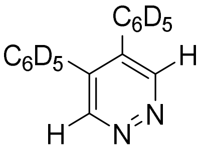

S7

A solution of di(d_5-phenyl)acetylene (145 mg, 0.96 mmol) and 3,6-dichloro-1,2,4,5-tetrazine (180 mg, 1.2 mmol, 1.2 eq.) in PhMe (4 mL) was heated to 180°C for 16 hours under microwave irradiation. The mixture was concentrated under reduced pressure and purified by flash column chromatography with 1:9 EtOAc:hexane as eluent to give 3,6-dichloro-4,5-di(d_5-phenyl)pyridazine **SXX** (108 mg, 36%) as a light pink solid; **^{13}C NMR** (101 MHz, $CDCl_3$) δ 156.3 (s), 141.7 (s), 132.8 (s), 128.9 (t, J = 24.3 Hz), 128.6 (t, J = 24.6 Hz), 127.9 (t, J = 24.5 Hz); **MS** (ESI) *m/z* 311 [(M + H)$^+$, 100]; **HRMS** (ESI) *m/z* [M + H]$^+$ calculated for $C_{16}HD_{10}N_2Cl_2{}^+$ 311.0921, found 311.0918 (+3.2 ppm error).

3-chloro-6-(d_3-methyl)-4,5-di(d_5-phenyl)pyridazine-4,5-$^{13}C_2$ S2

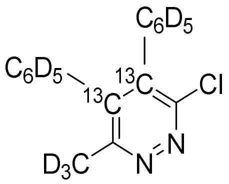

S2

To a solution of 3,6-dichloro-4,5-di(d_5-phenyl)pyridazine-4,5-$^{13}C_2$ **3** (30 mg, 0.096 mmol) and $Fe(acac)_3$ (3.5 mg, 9.9 µmol, 10 mol%) in THF (0.5 mL) and *N*-methyl-2-pyrrolidone (0.1 mL) at 0 °C was added d_3-methylmagnesium iodide solution (1.0 M in diethyl ether, 100 µL, 0.10 mmol, 1.05 eq.) dropwise. The reaction mixture was stirred at room temperature for 15 minutes, then diluted with water (10 mL). The mixture was extracted with ethyl acetate (3 × 10 mL), then washed with water (3 × 10 mL), brine (10 mL), dried over $MgSO_4$ and concentrated under reduced pressure. The product was purified by flash column chromatography with 4:1 petrol-EtOAc as eluent to give 3-chloro-4,5-di(d_5-phenyl)-6-(d_3-methyl)pyridazine-4,5-$^{13}C_2$ **S2** (12 mg, 42%) as a light brown solid; **^{13}C NMR** (101 MHz, $CDCl_3$) δ 141.7 (d, J = 57.0 Hz), 138.9 (d, J = 57.0 Hz); **MS** (ESI) *m/z* 296 [(M + H)$^+$, 100]; **HRMS** (ESI) *m/z* [M + H]$^+$ calculated for $C_{15}{}^{13}C_2HD_{13}N_2Cl^+$ 296.1723, found 296.1718 (+1.6 ppm error).

Comparison to 3-chloro-6-(d_3-methyl)-4,5-di(d_5-phenyl)pyridazine (S8) with carbon at natural abundance

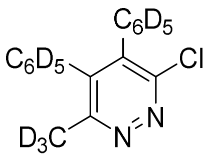

S8

To a solution of 3,6-dichloro-4,5-di(d_5-phenyl)pyridazine **S6** (30 mg, 0.096 mmol) and $Fe(acac)_3$ (3.5 mg, 9.9 µmol, 10 mol%) in THF (0.5 mL) and *N*-methyl-2-pyrrolidone (0.1 mL) at 0 °C was added d_3-methylmagnesium iodide solution (1.0 M in diethyl ether, 100 µL, 0.10 mmol, 1.05 eq.) dropwise. The reaction mixture was stirred at room temperature for 15 minutes, then diluted with water (10 mL). The mixture was extracted with ethyl acetate (3 × 10 mL), then washed with water (3 × 10 mL), brine (10 mL), dried over $MgSO_4$ and concentrated under reduced pressure. Purified by flash column chromatography with 4:1 petrol-EtOAc as eluent to give 3-chloro-4,5-di(d_5-phenyl)-6-(d_3-methyl)pyridazine **S8** (6 mg, 21%) as a colorless solid; **^{13}C NMR** (101 MHz, $CDCl_3$) δ 158.7 (s), 155.1 (s), 141.6 (s), 138.9 (s), 134.5 (s), 133.5 (s), 129.0 (t, J = 24.4 Hz), 128.4 (t, J = 24.3 Hz), 128.0 (t, J = 24.3 Hz), 128.0 (t, J = 24.4 Hz), 127.8 (t, J = 24.4 Hz), 127.6 (t, J = 24.3 Hz), 20.8 (sept, J = 19.8 Hz); **MS** (ESI) *m/z* 294 [(M + H)$^+$, 100; **HRMS** (ESI) *m/z* [M + H]$^+$ calculated for $C_{17}HD_{13}N_2Cl^+$ 294.1656, found 294.1649 (+3.4 ppm error).

3-(d_5-ethyl)-6-(d_3-methyl)-4,5-di(d_5-phenyl)pyridazine-4,5-$^{13}C_2$ 8

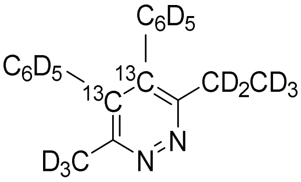

8

A solution of d_5-bromoethane (200 µL, 2.7 mmol) in THF (0.5 mL) was added dropwise to a suspension of magnesium turnings (65 mg, 2.7 mmol) in THF (3 mL), and the reaction mixture was stirred at room temperature for 1 hour. The resulting suspension was added slowly to a solution of 3-chloro-4,5-di(d_5-phenyl)-6-(d_3-methyl)pyridazine-4,5-$^{13}C_2$ **S2** (16 mg, 0.05 mmol) and Fe(acac)$_3$ (2.5 mg, 7.1 µmol, 15 mol%) in THF (0.5 mL) and NMP (0.1 mL) at 0 °C. The reaction mixture was stirred at room temperature for 20 minutes, then diluted with water (30 mL). The mixture was extracted with ethyl acetate (3 × 50 mL), then washed with water (3 × 50 mL), brine (20 mL), dried over $MgSO_4$ and concentrated under reduced pressure. The product was purified by flash column chromatography with EtOAc as eluent to give 3-(d_5-ethyl)-6-(d_3-methyl)-4,5-di(d_5-phenyl)pyridazine-4,5-$^{13}C_2$ **8** (10 mg, 63%) as a colorless solid; **^{13}C NMR** (101 MHz, $CDCl_3$) δ 139.0 (d, J = 57.3 Hz), 138.2 (d, J = 57.0 Hz); **MS** (ESI) m/z 295 [(M + H)$^+$, 100]; **HRMS** (ESI) m/z [M + H]$^+$ calculated for $C_{17}{}^{13}C_2HD_{18}N_2^+$ 295.2740, found 295.2739 (+0.1 ppm error).

Comparison to 3-(d_5-ethyl)-6-(d_3-methyl)-4,5-di(d_5-phenyl)pyridazine (S9) with carbon at natural abundance

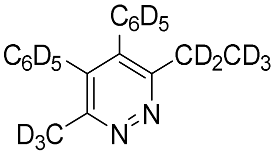

S9

A solution of d_5-bromoethane (200 µL, 2.7 mmol) in THF (0.5 mL) was added dropwise to a suspension of magnesium turnings (65 mg, 2.7 mmol) in THF (3 mL), and the reaction mixture was stirred at room temperature for 1 hour. The resulting suspension was added slowly to a solution of 3-chloro-4,5-di(d_5-phenyl)-6-(d_3-methyl)pyridazine **S8** (42 mg, 0.14 mmol) and Fe(acac)$_3$ (5 mg, 0.014 mmol, 10 mol%) in THF (1 mL) and NMP (0.2 mL) at 0 °C. The reaction mixture was stirred at room temperature for 10 minutes, then diluted with water (30 mL). The mixture was extracted with ethyl acetate (3 × 20 mL), then washed with water (3 × 20 mL), brine (10 mL), dried over $MgSO_4$ and concentrated under reduced pressure. The product was purified by flash column chromatography with EtOAc as eluent to give 3-(d_5-ethyl)-6-(d_3-methyl)-4,5-di(d_5-phenyl)pyridazine **S9** (28 mg, 68%) as a colorless solid; **^{13}C NMR** (101 MHz, $CDCl_3$) δ 161.1 (s), 156.6 (s), 138.9 (s), 138.3 (s), 135.5 (s), 135.2 (s), 127.7 (t, J = 24.6 Hz), 127.6 (t, J = 24.2 Hz), 127.1 (t, J = 24.6 Hz), 26.7 (pent, J = 19.1 Hz), 20.9 (sept, J = 19.8 Hz), 12.8 (sept, J = 19.6 Hz); **MS** (ESI) m/z 293 [(M + H)$^+$, 100]; **HRMS** (ESI) m/z [M + H]$^+$ calculated for $C_{19}HD_{18}N_2^+$ 293.2673, found 293.2672 (+0.4 ppm error).

3-chloro-6-*d*-4,5-di(*d*$_5$-phenyl)pyridazine-4,5-$^{13}C_2$ S3 and **3,6-*d*$_2$-4,5-di(*d*$_5$-phenyl)pyridazine-4,5-$^{13}C_2$ 2**

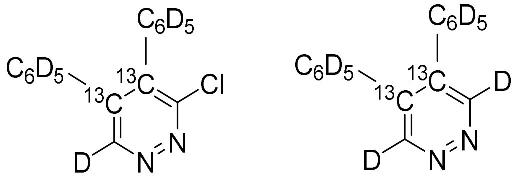

S3 **2**

To a suspension of 3,6-dichloro-4,5-di(*d*$_5$-phenyl)pyridazine-4,5-$^{13}C_2$ **3** (20 mg, 0.064 mmol), and K_2CO_3 (18 mg, 0.13 mmol, 2 eq.) in EtOD (10 mL) in a 30 mL Parr reactor was added 5% Pd/C (2 mg, 10 wt%). The reactor was sealed, purged with N_2, then pressurized with D_2 (8 bar). The reaction mixture was stirred at room temperature for 18 hours. The pressure was released and the suspension was filtered through Celite and washed with EtOH. The filtrate was concentrated under reduced pressure and purified by flash column chromatography with 1:1 to 1:9 hexane:EtOAc as eluent to give 3-chloro-6-*d*-4,5-di(*d*$_5$-phenyl)pyridazine-4,5-$^{13}C_2$ **S3** (5 mg, 28%) as a colorless solid; **^{13}C NMR** (101 MHz, $CDCl_3$) δ 140.8 (d, J = 56.7 Hz), 138.2 (d, J = 56.7 Hz); **MS** (ESI) *m/z* 280 [(M + H)$^+$, 100]; **HRMS** (ESI) *m/z* [M + H]$^+$ calculated for $C_{14}{}^{13}C_2HD_{11}N_2Cl^+$ 280.1441, found 280.1445 (+1.1 ppm error); and 3,6-*d*$_2$-4,5-di(*d*$_5$-phenyl)pyridazine-4,5-$^{13}C_2$ **2** (10 mg, 64%) as a colorless solid; **^{13}C NMR** (101 MHz, $CDCl_3$) δ 137.2 (s); **MS** (ESI) *m/z* 247 [(M + H)$^+$, 100]; **HRMS** (ESI) *m/z* [M + H]$^+$ calculated for $C_{14}{}^{13}C_2HD_{12}N_2{}^+$ 247.1894, found 247.1886 (+3.2 ppm error).

Comparison to 3-chloro-6-*d*-4,5-di(*d*$_5$-phenyl)pyridazine (S10) and **3,6-*d*$_2$-4,5-di(*d*$_5$-phenyl)pyridazine (S11) with carbon at natural abundance**

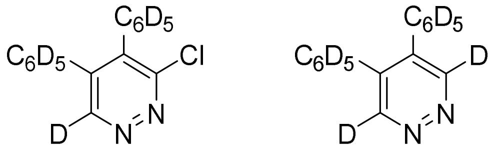

S10 **S11**

To a suspension of 3,6-dichloro-4,5-di(*d*$_5$-phenyl)pyridazine **S6** (20 mg, 0.064 mmol), and K_2CO_3 (18 mg, 0.13 mmol, 2 eq.) in EtOD (10 mL) in a 30 mL Parr reactor was added 5% Pd/C (2 mg, 10 wt%). The reactor was sealed, purged with N_2, then pressurized with D_2 (8 bar). The reaction mixture was stirred at room temperature for 4 hours. The pressure was released and the suspension was filtered through Celite and washed with EtOH. The filtrate was concentrated under reduced pressure and purified by flash column chromatography with 1:1 to 1:9 hexane:EtOAc as eluent to give 3-chloro-6-*d*-4,5-di(*d*$_5$-phenyl)pyridazine **S10** (6 mg, 34%) as a colorless solid; **^{13}C NMR** (101 MHz, $CDCl_3$) δ 157.1 (s), 151.3 (t, J = 28.0 Hz), 140.8 (s), 138.3 (s), 133.9 (s), 133.0 (s), 129.4 (t, J = 24.5 Hz), 129.0 (t, J = 24.3 Hz), 128.7 (t, J = 25.2 Hz), 128.6 (t, J = 23.6 Hz), 128.3 (t, J = 24.5 Hz), 128.1 (t, J = 24.3 Hz); **MS** (ESI) *m/z* 278 [(M + H)$^+$, 100], 280 (42); **HRMS** (ESI) *m/z* [M + H]$^+$ calculated for $C_{16}HD_{11}N_2Cl^+$ 278.1374, found 278.1370 (+2.2 ppm error); and 3,6-*d*$_2$-4,5-di(*d*$_5$-phenyl)pyridazine **S11** (6 mg, 38%) as a colourless solid; **^{13}C NMR** (101 MHz, $CDCl_3$) δ 151.8 (t, J = 27.8 Hz), 137.3 (s), 134.6 (s), 129.1 (t, J = 24.4 Hz), 128.5 (t, J = 24.1 Hz), 128.5 (t, J = 24.6 Hz); **MS** (ESI) *m/z* 245 [(M + H)$^+$, 100]; **HRMS** (ESI) *m/z* [M + H]$^+$ calculated for $C_{16}HD_{12}N_2{}^+$ 245.1826, found 245.1823 (+1.6 ppm error).

3-*d*-4,5-di(*d*$_5$-phenyl)pyridazine-4,5-$^{13}C_2$ 9

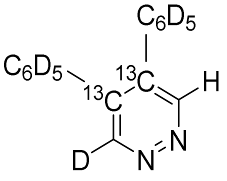

9

To a suspension of 3-chloro-6-*d*-4,5-di(*d*$_5$-phenyl)pyridazine-4,5-$^{13}C_2$ **S3** (5 mg, 0.018 mmol), and K_2CO_3 (5 mg, 0.04 mmol, 2 eq.) in EtOH (3 mL) in a 30 mL Parr reactor was added 5% Pd/C (1 mg, 20 wt%). The reactor was sealed, purged with N_2, then pressurized with H_2 (8 bar). The reaction mixture was stirred at room temperature for 2 hours. The pressure was released and the suspension was filtered through Celite and washed with EtOH. The filtrate was concentrated under reduced pressure and purified by flash column chromatography with EtOAc as eluent to give 3-*d*-4,5-di(*d*$_5$-phenyl)pyridazine-4,5-$^{13}C_2$ **9** (3 mg, 68%) as a light brown solid; **^{1}H NMR** (400 MHz, $CDCl_3$) δ 9.21 (dd, J = 5.32 Hz, 5.32 Hz); **^{13}C NMR** (101 MHz, $CDCl_3$) δ 137.3 (s); **MS** (ESI) *m/z* 246 [(M + H)$^+$, 100]; **HRMS** (ESI) *m/z* [M + H]$^+$ calculated for $C_{14}{}^{13}C_2H_2D_{11}N_2^+$ 246.1831, found 246.1819 (+4.3 ppm error).

Comparison to 3-*d*-4,5-di(*d*$_5$-phenyl)pyridazine (S12) with carbon at natural abundance

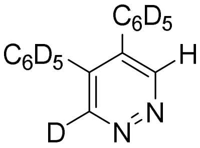

S12

To a suspension of 3-chloro-6-*d*-4,5-di(*d*$_5$-phenyl)pyridazine **S10** (6 mg, 0.022 mmol), and K_2CO_3 (5 mg, 0.04 mmol, 2 eq.) in EtOH (3 mL) in a 30 mL Parr reactor was added 5% Pd/C (1 mg, 20 wt%). The reactor was sealed, purged with N_2, then pressurised with H_2 (8 bar). The reaction mixture was stirred at room temperature for 24 hours. The pressure was released and the suspension was filtered through Celite and washed with EtOH. The filtrate was concentrated under reduced pressure and purified by flash column chromatography with EtOAc as eluent to give 3-*d*-4,5-di(*d*$_5$-phenyl)pyridazine **S12** (4 mg, 75%) as a colourless solid.

1-(d_7-*p*-tolyl)-2-(d_5-phenyl)acetylene-1,2-$^{13}C_2$ S5

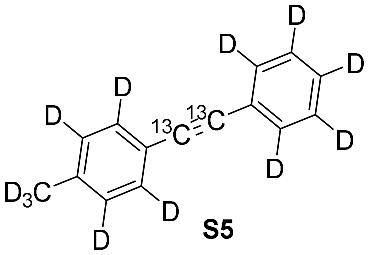

S5

A solution of 4-bromotoluene-d_7 **S4** (250 mg, 1.5 mmol), (trimethylsilyl)acetylene-$^{13}C_2$ (210 µL, 1.5 mmol) and bis(triphenylphosphine)palladium(II) dichloride (50 mg, 0.071 mmol, 5 mol%) in piperidine (2 mL) was heated to 70°C for 18 hours under microwave irradiation. The reaction mixture was diluted with water (20 mL) and extracted with dichloromethane (3 × 20 mL). The combined organic extracts were washed with water (20 mL), dried over $MgSO_4$ and concentrated under reduced pressure. Passed through a plug of silica, washing with hexane (50 mL), and concentrated under reduced pressure to give a colorless oil (166 mg). This residue was dissolved in piperidine (2 mL), and bromobenzene-d_5 (150 µL, 1.4 mmol) and bis(triphenylphosphine)palladium(II) dichloride (26 mg, 0.037 mmol, 5 mol%) were added. Tetra-*n*-butylammonium fluoride solution (1.0 M in THF, 0.77 mL, 0.77 mmol) was added and the reaction mixture was stirred at room temperature for 10 minutes, then heated to 70°C for 19 hours under microwave irradiation. The reaction mixture was diluted with water (20 mL) and extracted with dichloromethane (3 × 20 mL). The combined organic extracts were washed with water (20 mL), dried over $MgSO_4$ and concentrated under reduced pressure. Purified by flash column chromatography with hexane as eluent to give 1-(d_7-*p*-tolyl)-2-(d_5-phenyl)acetylene-1,2-$^{13}C_2$ **S5** (105 mg, 38%) as a colorless solid; **^{13}C NMR** (101 MHz, $CDCl_3$) δ 89.3 (app. s), 89.1 (app. s).

Comparison to 1-(d_7-*p*-tolyl)-2-(d_5-phenyl)acetylene (S13) with carbon at natural abundance

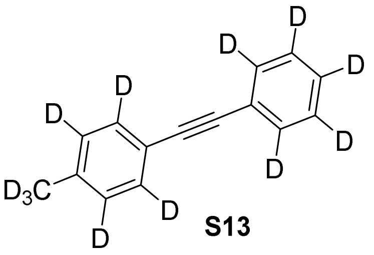

S13

A solution of 4-bromotoluene-d_7 **S4** (250 mg, 1.5 mmol), (trimethylsilyl)acetylene (210 µL, 1.5 mmol) and bis(triphenylphosphine)palladium(II) dichloride (50 mg, 0.071 mmol, 5 mol%) in piperidine (2 mL) was heated to 70°C for 18 hours under microwave irradiation. The reaction mixture was diluted with water (20 mL) and extracted with dichloromethane (3 × 20 mL). The combined organic extracts were washed with water (20 mL), dried over $MgSO_4$ and concentrated under reduced pressure. Passed through a plug of silica, washing with hexane (50 mL), and concentrated under reduced pressure to give a colorless oil (170 mg). This residue was dissolved in piperidine (2 mL), and bromobenzene-d_5 (150 µL, 1.4 mmol) and bis(triphenylphosphine)palladium(II) dichloride (26 mg, 0.037 mmol, 5 mol%) were added. Tetra-*n*-butylammonium fluoride solution (1.0 M in THF, 0.77 mL, 0.77 mmol) was added and the reaction mixture was stirred at room temperature for 10 minutes, then heated to 70°C for 19 hours under microwave irradiation. The reaction mixture was diluted with water (20 mL) and extracted with dichloromethane (3 × 20 mL). The combined organic extracts were washed with water (20 mL), dried over $MgSO_4$ and concentrated under reduced pressure. The product was purified by flash column chromatography with hexane as eluent to give 1-(d_7-*p*-tolyl)-2-(d_5-phenyl)acetylene **S13** (210 mg, 69%) as a colorless solid; **^{13}C NMR** (101 MHz, $CDCl_3$) δ 138.2 (s), 131.24 (t, J = 24.8 Hz), 131.20 (t, J =

24.8 Hz), 128.8 (t, J = 24.0 Hz), 127.9 (t, J = 25.3 Hz), 127.7 (t, J = 25.3 Hz), 123.4 (s), 120.1 (s), 89.7 (s), 88.8 (s), 20.7 (sept, J = 19.4 Hz).

3,6-dichloro-4-(d_7-*p*-tolyl)-5-(d_5-phenyl)pyridazine-4,5-$^{13}C_2$ 5

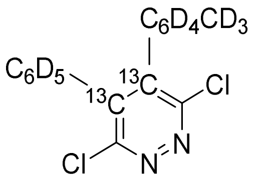

5

A solution of 1-(d_7-*p*-tolyl)-2-(d_5-phenyl)acetylene-1,2-$^{13}C_2$ **S5** (50 mg, 0.24 mmol) and 3,6-dichloro-1,2,4,5-tetrazine (37 mg, 0.25 mmol, 1 eq.) in PhMe (2 mL) was heated to 180°C for 16 hours under microwave irradiation. The mixture was concentrated under reduced pressure and purified by flash column chromatography with 1:9 EtOAc:hexane as eluent to give 3,6-dichloro-4-(d_7-*p*-tolyl)-5-(d_5-phenyl)pyridazine-4,5-$^{13}C_2$ **5** (16 mg, 28%) as a colorless solid; **^{13}C NMR** (101 MHz, $CDCl_3$) δ 141.73 (app s), 141.72 (app s); **MS** (ESI) *m/z* 329 [(M + H)$^+$, 100], 331 (87); **HRMS** (ESI) *m/z* [M + H]$^+$ calculated for $C_{15}{}^{13}C_2Cl_2HD_{12}N_2^+$ 329.1271, found 329.1268 (+5.0 ppm error).

Comparison to 3,6-dichloro-4-(d_7-*p*-tolyl)-5-(d_5-phenyl)pyridazine (S14) with carbon at natural abundance

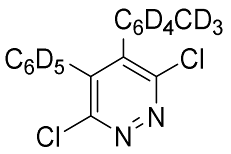

S14

A solution of 1-(d_7-*p*-tolyl)-2-(d_5-phenyl)acetylene **S13** (100 mg, 0.48 mmol) and 3,6-dichloro-1,2,4,5-tetrazine (74 mg, 0.49 mmol, 1 eq.) in PhMe (2 mL) was heated to 180°C for 16 hours under microwave irradiation. The mixture was concentrated under reduced pressure and purified by flash column chromatography with 1:9 EtOAc:hexane as eluent to give 3,6-dichloro-4-(d_7-*p*-tolyl)-5-(d_5-phenyl)pyridazine **S14** (56 mg, 36%) as a light pink solid; **^{13}C NMR** (101 MHz, $CDCl_3$) δ 156.5 (s), 156.2 (s), 141.8 (s), 141.7 (s), 138.9 (s), 133.0 (s), 129.8 (s), 128.9 (t, J = 24.7 Hz), 128.8 (t, J = 24.2 Hz), 128.7 (t, J = 24.9 Hz), 128.5 (t, J = 24.3 Hz), 127.9 (t, J = 24.5 Hz), 20.5 (sept, J = 19.4 Hz); **MS** (ESI) *m/z* 327 [(M + H)$^+$, 100]; **HRMS** (ESI) *m/z* [M + H]$^+$ calculated for $C_{17}Cl_2HD_{12}N_2^+$ 327.1204, found 327.1196 (+4.6 ppm error).

3,6-d_2-4-(d_7-*p*-tolyl)-5-(d_5-phenyl)pyridazine-4,5-$^{13}C_2$ 4

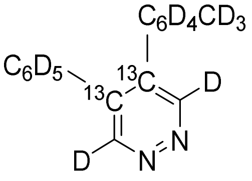

4

To a suspension of 3,6-dichloro-4-(d_7-*p*-tolyl)-5-(d_5-phenyl)pyridazine-4,5-$^{13}C_2$ **5** (12 mg, 0.036 mmol), and K_2CO_3 (13 mg, 0.094 mmol, 2.5 eq.) in EtOD (5 mL) in a 30 mL Parr reactor was added 5% Pd/C (2 mg). The reactor was sealed, purged with N_2, then pressurized with D_2 (8 bar). The reaction mixture was stirred at room temperature for 1 hour. The pressure was released and the suspension was filtered through Celite and washed with EtOH. The filtrate was concentrated under reduced pressure and purified by flash column chromatography with EtOAc as eluent to give 3,6-d_2-4-(d_7-*p*-tolyl)-5-(d_5-phenyl)pyridazine-4,5-$^{13}C_2$ **4** (9 mg, 95%) as a colorless solid; **^{13}C NMR** (101 MHz, $CDCl_3$) δ 137.2 (s); **MS** (ESI) *m/z* 263 [(M + H)$^+$, 100]; **HRMS** (ESI) *m/z* [M + H]$^+$ calculated for $C_{15}{}^{13}C_2HD_{14}N_2^+$ 263.2176, found 263.2164 (+4.6 ppm error).

Comparison to 3,6-d_2-4-(d_7-*p*-tolyl)-5-(d_5-phenyl)pyridazine (S15) with carbon at natural abundance

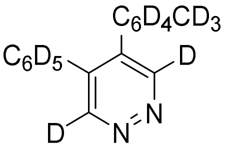

S15

To a suspension of 3,6-dichloro-4-(d_7-*p*-tolyl)-5-(d_5-phenyl)pyridazine **S13** (15 mg, 0.057 mmol), and K_2CO_3 (17 mg, 0.12 mmol, 2 eq.) in EtOD (5 mL) in a 30 mL Parr reactor was added 5% Pd/C (2 mg). The reactor was sealed, purged with N_2, then pressurized with D_2 (8 bar). The reaction mixture was stirred at room temperature for 4 hours. The pressure was released and the suspension was filtered through Celite and washed with EtOH. The filtrate was concentrated under reduced pressure and purified by flash column chromatography with EtOAc as eluent to give 3,6-d_2-4-(d_7-*p*-tolyl)-5-(d_5-phenyl)pyridazine **S15**.

3,6-di(d_3-methoxy)-4-(d_7-*p*-tolyl)-5-(d_5-phenyl)pyridazine-4,5-$^{13}C_2$ 6

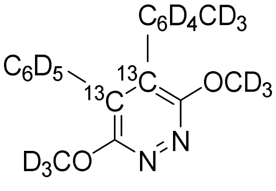

6

To a solution of 3,6-dichloro-4-(d_7-*p*-tolyl)-5-(d_5-phenyl)pyridazine-4,5-$^{13}C_2$ **5** (10 mg, 0.030 mmol) in methanol-d_4 (2 mL) was added sodium hydride (25 mg, 60% suspension in mineral oil, 0.63 mmol, 20 eq.) and the reaction mixture was stirred at reflux for 24 hours. Quenched by addition of saturated NH_4Cl solution (10 mL), extracted with EtOAc (3 × 10 mL), dried over $MgSO_4$ and concentrated under reduced pressure. The product was purified by flash column chromatography with 1:9 EtOAc:hexane as eluent to give 3,6-di(d_3-methoxy)-4-(d_7-*p*-tolyl)-5-(d_5-phenyl)pyridazine-4,5-$^{13}C_2$ **6** (10 mg, 100%) as a colorless solid; **^{13}C NMR** (101 MHz, $CDCl_3$) *δ* 132.1 (app s), 132.0 (app s); **MS** (ESI) *m/z* 327 [(M + H)$^+$, 100]; **HRMS** (ESI) *m/z* [M + H]$^+$ calculated for $C_{17}{}^{13}C_2HD_{18}N_2O_2^+$ 327.2638, found 327.2625 (+3.9 ppm error).

Comparison to 3,6-di(d_3-methoxy)-4-(d_7-*p*-tolyl)-5-(d_5-phenyl)pyridazine (S16) with carbon at natural abundance

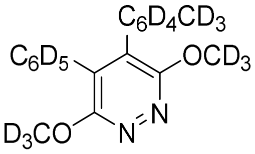

S16

To a solution of 3,6-dichloro-4-(d_7-*p*-tolyl)-5-(d_5-phenyl)pyridazine **S14** (10 mg, 0.030 mmol) in methanol-d_4 (2 mL) was added sodium hydride (25 mg, 60% suspension in mineral oil, 0.63 mmol, 20 eq.) and the reaction mixture was stirred at reflux for 3 days. Quenched by addition of saturated NH_4Cl solution (10 mL), extracted with EtOAc (3 × 10 mL), dried over $MgSO_4$ and concentrated under reduced pressure. Purified by flash column chromatography with 1:9 EtOAc:hexane as eluent to give 3,6-di(d_3-methoxy)-4-(d_7-*p*-tolyl)-5-(d_5-phenyl)pyridazine **S16** (6 mg, 62%) as a colorless solid; **^{13}C NMR** (101 MHz, $CDCl_3$) *δ* 160.4 (s), 160.3 (s), 137.7 (s), 132.5 (s), 132.1 (s), 131.9 (s), 129.9 (t, *J* = 24.3 Hz), 129.8 (t, *J* = 24.3 Hz), 129.2 (s), 128.3 (t, *J* = 23.9 Hz), 127.5 (t, *J* = 24.6 Hz), 127.4 (t, *J* = 24.4 Hz), 54.3 (sept, *J* = 22.1 Hz), 54.2 (sept, *J* = 22.1 Hz), 20.5 (sept, *J* = 19.2 Hz); **MS** (ESI) *m/z* 325 [(M + H)$^+$, 100]; **HRMS** (ESI) *m/z* [M + H]$^+$ calculated for $C_{19}HD_{18}N_2O_2^+$ 235.2571, found 325.2564 (+1.8 ppm error).

3,6-di(d_3-methyl)-4-(d_7-*p*-tolyl)-5-(d_5-phenyl)pyridazine-4,5-$^{13}C_2$ 7

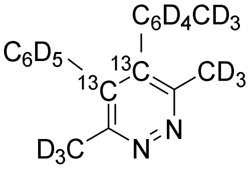

7

To a solution of 3,6-dichloro-4-(d_7-*p*-tolyl)-5-(d_5-phenyl)pyridazine-4,5-$^{13}C_2$ **5** (40 mg, 0.12 mmol) and Fe(acac)$_3$ (4 mg, 0.01 mmol, 10 mol%) in THF (1.5 mL) and *N*-methyl-2-pyrrolidone (0.25 mL) at 0 °C was added d_3-methylmagnesium iodide solution (1.0 M in diethyl ether, 270 µL, 0.27 mmol, 2.3 eq.) dropwise. The reaction mixture was stirred at room temperature for 10 minutes, then diluted with water (10 mL). The mixture was extracted with ethyl acetate (3 × 10 mL), then washed with water (3 × 10 mL), brine (10 mL), dried over $MgSO_4$ and concentrated under reduced pressure. The product was purified by flash column chromatography with EtOAc as eluent to give 3,6-di(d_3-methyl)-4-(d_7-*p*-tolyl)-5-(d_5-phenyl)pyridazine-4,5-$^{13}C_2$ **7** (31 mg, 88%) as a colorless solid; **^{13}C NMR** (101 MHz, $CDCl_3$) δ 138.7 (s); **MS** (ESI) *m/z* 294 [(M + H)$^+$, 100]; **HRMS** (ESI) *m/z* [M + H]$^+$ calculated for $C_{17}{}^{13}C_2HD_{18}N_2^+$ 295.2740, found 295.2746 (–1.9 ppm error).

Comparison to 3,6-di(d_3-methyl)-4-(d_7-*p*-tolyl)-5-(d_5-phenyl)pyridazine (S17) with carbon at natural abundance

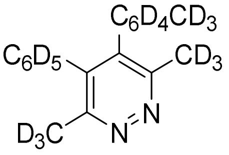

S17

To a solution of 3,6-dichloro-4-(d_7-*p*-tolyl)-5-(d_5-phenyl)pyridazine **S14** (20 mg, 0.062 mmol) and Fe(acac)$_3$ (2 mg, 0.005 mmol, 10 mol%) in THF (0.75 mL) and *N*-methyl-2-pyrrolidone (0.15 mL) at 0 °C was added d_3-methylmagnesium iodide solution (1.0 M in diethyl ether, 140 µL, 0.14 mmol, 2.3 eq.) dropwise. The reaction mixture was stirred at room temperature for 10 minutes, then diluted with water (10 mL). The mixture was extracted with ethyl acetate (3 × 10 mL), then washed with water (3 × 10 mL), brine (10 mL), dried over $MgSO_4$ and concentrated under reduced pressure. Purified by flash column chromatography with EtOAc as eluent to give 3,6-di(d_3-methyl)-4-(d_7-*p*-tolyl)-5-(d_5-phenyl)pyridazine **S17** (12 mg, 67%) as a colorless solid; **^{13}C NMR** (101 MHz, $CDCl_3$) δ 157.1 (s), 156.8 (s), 138.8 (s), 138.7 (s), 137.2 (s), 135.6 (s), 132.4 (s), 128.63 (t, *J* = 24.4 Hz), 128.56 (t, *J* = 24.3 Hz), 128.5 (t, *J* = 24.3 Hz), 127.8 (t, *J* = 24.3 Hz), 127.2 (t, *J* = 24.1 Hz), 20.8 (sept, *J* = 19.2 Hz), 20.4 (sept, *J* = 19.2 Hz); **MS** (ESI) *m/z* 293 [(M + H)$^+$, 100]; **HRMS** (ESI) *m/z* [M + H]$^+$ calculated for $C_{19}HD_{18}N_2^+$ 293.2673, found 293.2670 (+1.2 ppm error).

S4. Theory and simulations

In this work, we use standard density matrix based numerical approaches to study the SABRE process. The complete method has been extensively presented in earlier works.[7-8]

The whole catalytic process can be thought of as a three-step time dependent evolution process as described in the figure below. Step 1: evolution of the full spin system including *p*-H_2 at a defined mixing field; Step 2: evolution of the substrate spins after its dissociation from the catalyst; Step 3: evolution of substrate spins during time dependent magnetic field transfer while transporting the sample in to the spectrometer.

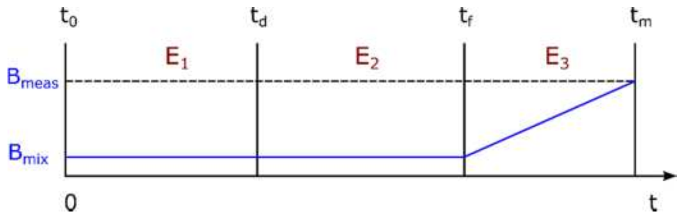

Figure S3. Magnetic field variance during the SABRE process. E_1, E_2, and E_3 represent three evolution periods during time intervals (t_0-t_d), (t_d-t_f), and (t_f-t_m) respectively. B_{mix} and B_{meas} represent the mixing (transfer) and measurement fields respectively.

After going through the complete procedure with common approximations, the final density matrix that results for a 2-spin substrate in the measurement field can be written as follows:

$$\bar{\rho}_m(t_m) = A * \hat{R}_z + B * \hat{T}_z + C * \hat{R}_z\hat{T}_z + \cdots \qquad \text{(S1)}$$

where $\hat{R}_z$ and $\hat{T}_z$ represent the longitudinal magnetization terms of two spins (*R* and *T*) in the substrate. The $\hat{R}_z\hat{T}_z$ term represents a double quantum term. Apart from these three terms, there will be several cross and higher order terms but their contributions are negligible in most of the cases and hence have been neglected in this study. A, B, and C are constant terms and their values actually reflect the enhancement factor over the thermal signal.

Appropriate routines in Mathematica were used to perform the calculations. The input parameters used in these calculations are taken from the related experimental data that is summarized in Table S1. Figure S3-S5 show simulations of SABRE derived magnetization at the indicated mixing field strength for two types of substrates. Despite this simplified treatment, the experimental data (Fig. S4-S6) shows good agreement with the results of simulation.

Table S1: Parameters used in the numerical calculations for substrate 1.

Spin System	Parameters		Values (ν_{1H} = 400 MHz) (ν_{13C} = 100 MHz)
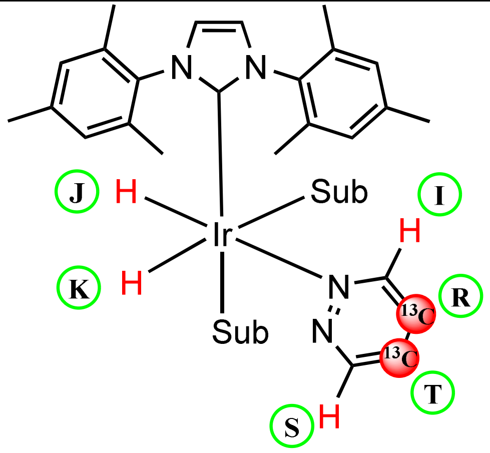 *Type-1:* **1**	***Chemical shifts (ppm)***	δ_J	-21.15 ppm (^{1}H)
		δ_K	-21.15 ppm (^{1}H)
		δ_I	9.24 ppm (^{1}H)
		δ_S	9.24 ppm (^{1}H)
		δ_R	138.5 ppm (^{13}C)
		δ_T	138.5 ppm (^{13}C)
	J-coupling constants (Hz)	J_{IS}	0.0 Hz
		J_{IR}	6.8 Hz
		J_{IT}	3.7 Hz
		J_{SR}	3.7 Hz
		J_{IS}	6.8 Hz
		J_{IR}	58.5 Hz
		J_{JK}	-8.0 Hz
		J_{JI}	0.9
		J_{JS}	0.1 Hz
		J_{JR}	0.15 Hz
		J_{JT}	0.05 Hz
		J_{KI}	0.1
		J_{KS}	0.02 Hz
		J_{KR}	0.05 Hz
		J_{KT}	0.01 Hz
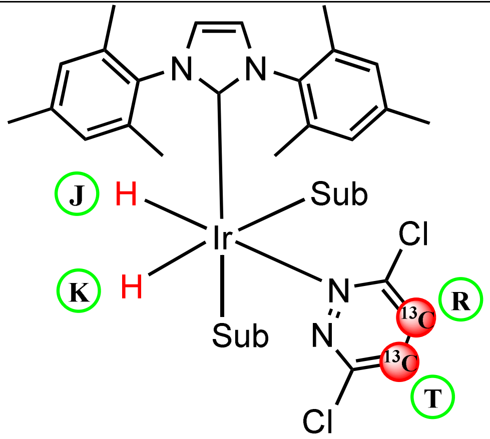 *Type-2:* **5**	***Chemical shifts (ppm)***	δ_J	-22.36 ppm (^{1}H)
		δ_K	-22.36 ppm (^{1}H)
		δ_R	142.4 ppm (^{13}C)
		δ_T	142.4 ppm (^{13}C)
	J-coupling constants (Hz)	J_{JK}	-8.0 Hz
		J_{JR}	0.15 Hz
		J_{JT}	0.05 Hz
		J_{KR}	0.05 Hz
		J_{KT}	0.01 Hz
		J_{RT}	58.5 Hz

Agent **1**, reflects an isolated 4-spin coupled system that becomes a 6-spin coupled spin network when it binds to the catalyst. The spin parameters are summarized in Table S1. The complexity of its spin dynamics as a function of mixing field is reflected in Figures S4-S6. The terms I_zR_z, I_zT_z, S_zT_z, S_zR_z, R_z and T_z will lead to observable signal when a single 90° pulse is applied at ^{13}C for detection as shown in Figure S4. However, if we apply simultaneous 90° pulses to both nuclei on the ^{1}H and ^{13}C channels for detection, then we only observe the terms R_Z and T_Z terms whilst filtering out the other terms (a de facto double-quantum filter). Figure S5 illustrates the success of this approach of filtration. It should be noted that in this study we primarily focused on polarizing single quantum coherences in order to apply the subsequent LLS sequence, hence all other cross terms have been filtered out during the process.

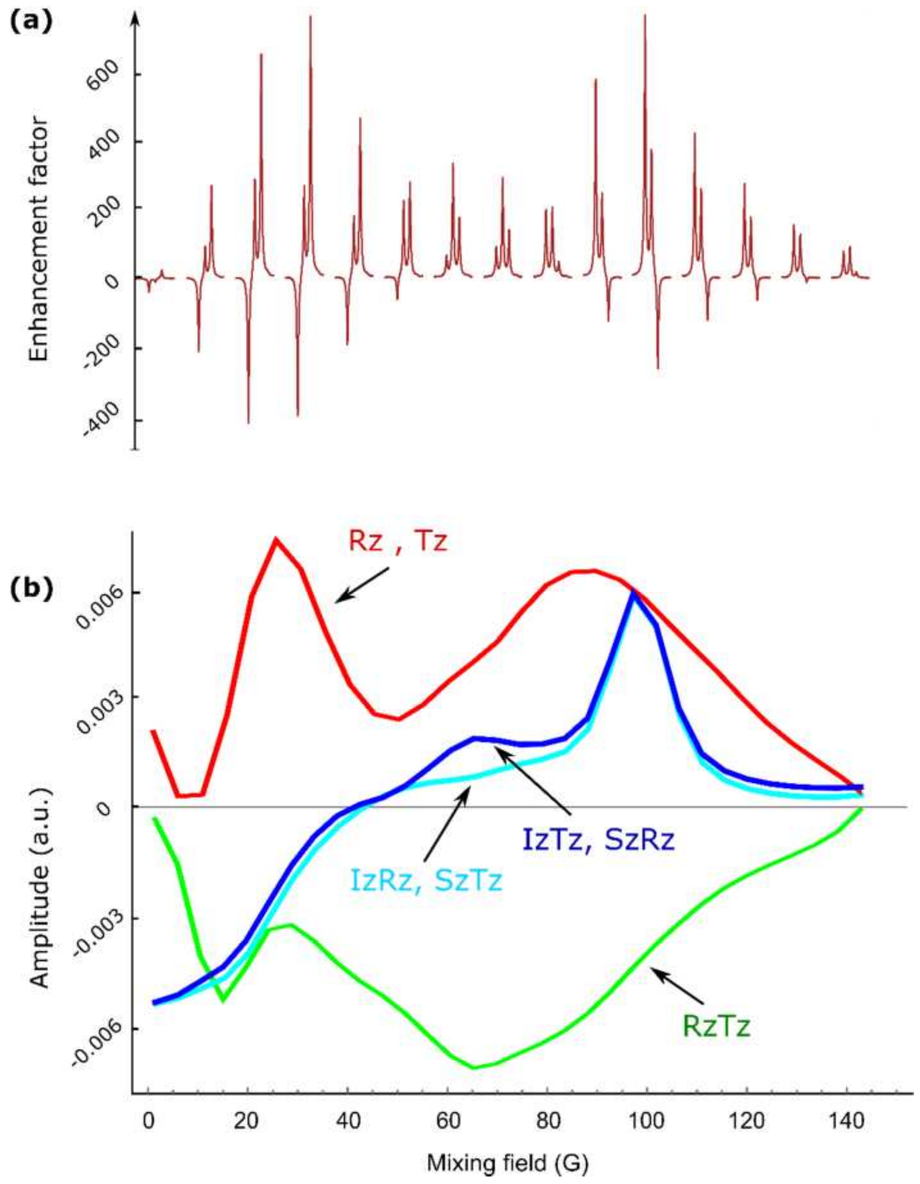

*Figure S4. (a) ^{13}C NMR SABRE derived response after a 90° pulse (applied only on ^{13}C channel) portrayed as the experimentally derived enhancement factor based on relaxed magnetization for **1** as a function of mixing field (Gauss) and (b) the corresponding simulated plot derived by the theoretical approach described above for the indicated terms produced in the mixing field. The terms I_zR_z, I_zT_z, S_zT_z, S_zR_z, R_z and T_z will lead to observable signal.*

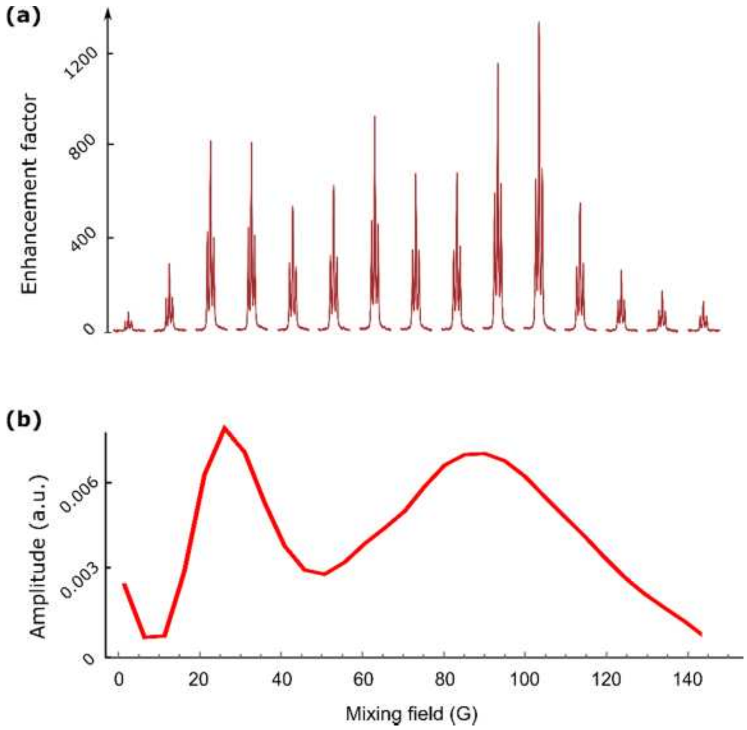

*Figure S5. (a) ^{13}C NMR SABRE derived response after 90° pulse (applied on both ^{13}C and ^{1}H channels) portrayed as the experimentally derived enhancement factor based on relaxed magnetization for **1** as a function of mixing field (Gauss) and (b) the corresponding simulated plot for R_Z + T_Z magnetization derived by the theoretical approach described above.*

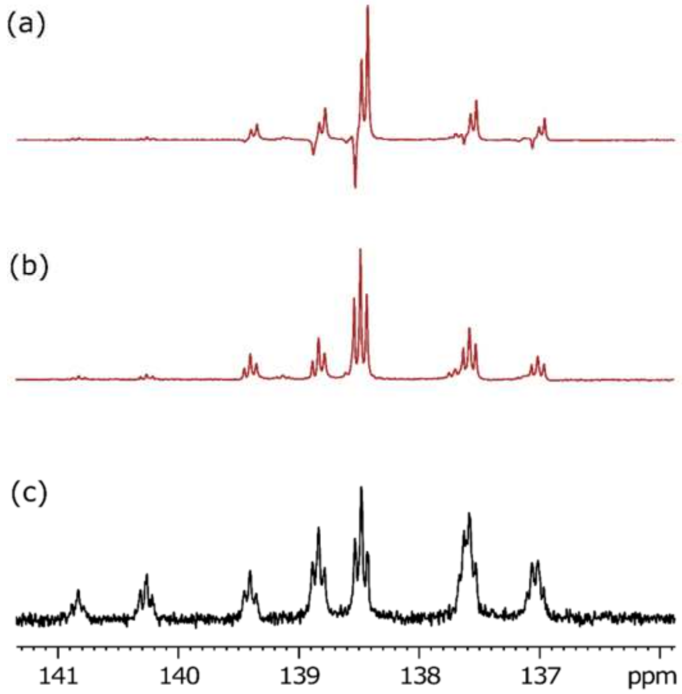

Figure S6. ^{13}C NMR SABRE derived response at 30 G after (a) 90° pulse applied only on the ^{13}C channel; (b) 90° pulse applied simultaneously on the ^{13}C and ^{1}H channels; (c) thermally polarized spectrum of 1000 transients for comparison. In the case of (a) major contributions to the signal originate from the terms I_zR_z, I_zT_z, S_zT_z, S_zR_z, R_z and T_z. While in the case of (b), only R_z and T_z lead to observable signal.

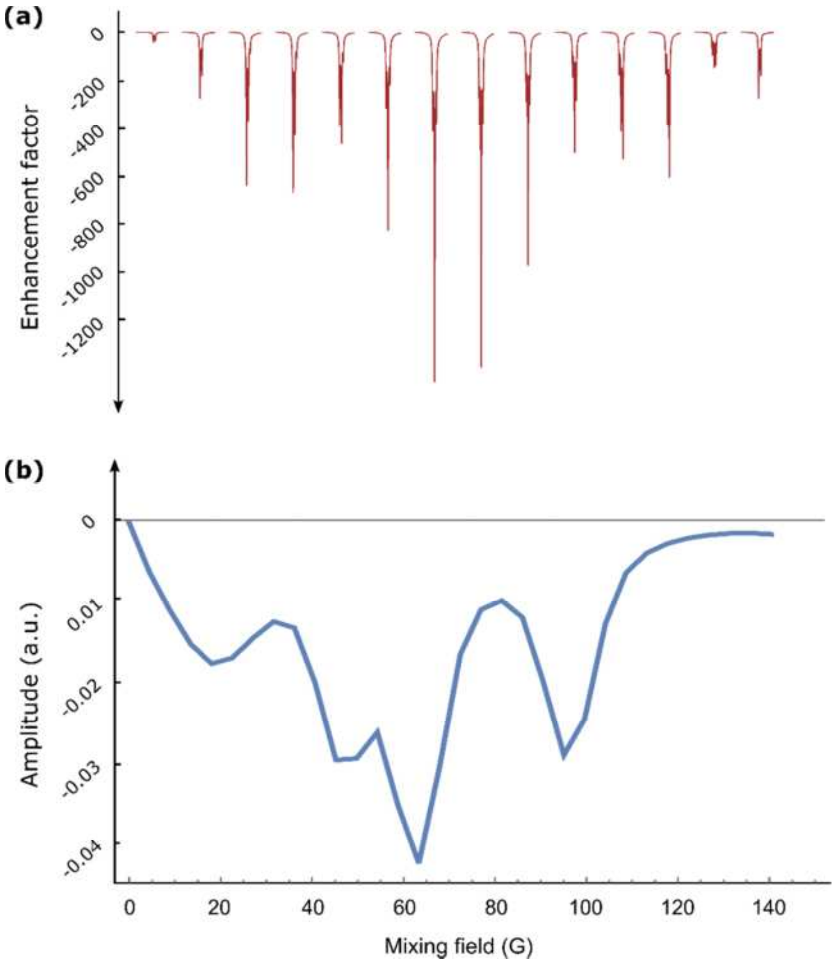

*Figure S7. (a) ^{1}H SABRE NMR signals of **1**, with relative signal levels after a 90° pulse (applied simultaneously on both ^{1}H and ^{13}C channels), as a function of mixing field and (b) corresponding simulated plot (sum of I_Z and S_Z terms) derived by the theoretical approach described above that will lead to observable signal. A maximum signal enhancement was observed for a 65 Gauss mixing field.*

Agents 4-8, will polarize via the direct mode of transfer as there are no suitable relay mechanisms on offer. This scenario significantly reduces the complexity of the spin dynamics. Almost the entire contribution to the observed signal now results from the single quantum R_Z, T_Z terms with very little of the double quantum term R_ZT_Z. The experimental field transfer plot closely matches with the theoretical simulations as shown in Figure S8.

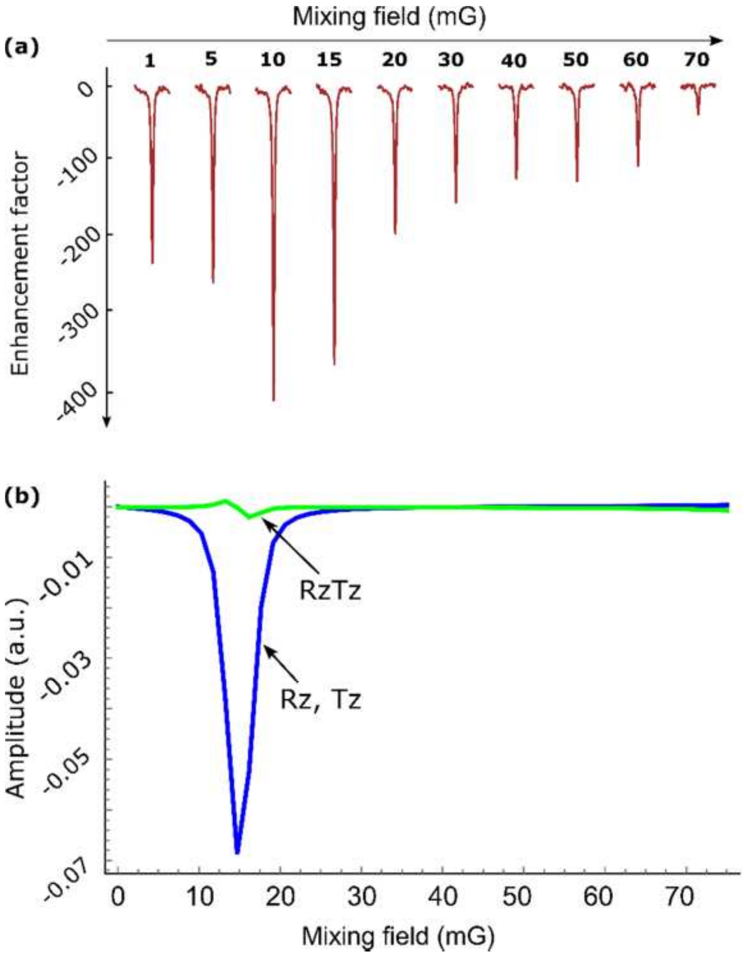

*Figure S8. (a) ^{13}C NMR SABRE derived response after a 90° pulse portrayed as the experimentally derived enhancement factor based on relaxed magnetization for **5** as a function of magnetic mixing field (Gauss) and (b) the corresponding simulated plot derived by the theoretical approach described above for the indicated terms produced in the mixing field. The terms R_z and T_z will lead to observable signal.*

The resonance condition for the direct mode of polarization transfer can also be verified through calculation as outlined by Theis *et* al. in their recent paper.[9]

$$B_{Resonance} \approx {J_{HH} - \frac{1}{2}J_{CH}} \Big/ {(\gamma_H - \gamma_C)}$$

where J_{HH} and J_{CH} are the hydride J-coupling constant and hydride-to-^{13}C coupling constant in Hz respectively. γ_H and γ_C represent gyromagnetic ratios of ^{1}H and ^{13}C nuclei respectively. Using the equation above, the theoretical estimation for optimum polarization transfer to the ^{13}C nuclei is these samples are found to be $\pm 0.3\ \mu$T (3 mG). This calculation, however, does not include several other important factors (e.g. residence time, exchange rates) required to make it precise. Experimentally, we found maxima in the range of 1 mG – 20 mG in all the cases.

S5. Long-lived singlet states (LLS)

A pair of coupled spin-1/2 particles can be defined by the total spin of the system. This can takes value of 1, in which case there are three possible quantum state degeneracies, commonly known as triplet states (analogous to those found in the ortho forms of dihydrogen molecule). The other possibility is that the total spin is 0, representing a non-magnetic state termed a singlet state, analogous to the para isomer of dihydrogen. In a coupled spin-1/2 system where both spins are chemically equivalent (e.g. H_2 molecule), the singlet and triplets forms correspond to exact Eigen states of the spin Hamiltonian and formation of the singlet state occurs naturally. However, it is not possible to access the singlet state (spin 0 = non-magnetic) without performing a symmetry breaking process. In 2004, it was first shown by Levitt and co-workers that it's possible to create similar singlet states in chemically inequivalent spin-1/2 pair via suitable radio frequency (*rf*) pulse sequencing. Since the singlet can be created experimentally, it can also be retrieved on demand via similar *rf* pulse sequencing. The importance of singlet state in NMR is huge considering the fact that it's unaffected by

one of the major relaxation mechanism. Consequently, a singlet state lifetime can be much longer than the standard nuclear spin lifetime that is dictated by T_1/T_2 relaxation time constants.

In this study, we used singlet state as the polarization bank. At first we create accessible hyperpolarized spin-order that is then converted into singlet order to preserve its lifetime. When required, the stored hyper-singlet states are converted back into an observable mode for detection. We used standard *rf* pulse sequencing protocols to create the hyper-singlet states as described below.

The M2S-S2M pulse sequence is among the most elegant to study long-lived singlet states (LLS), shown in Figure S9. Here M2S stands for 'magnetization-to-singlet' which converts magnetization into singlet states in an adiabatic fashion by applying a number of π pulses spaced over an optimized duration of time. After M2S, a variable waiting times were applied (τ_{delay}) to measure the singlet state decay time constant (T_S). During the waiting period a reverse order pulse S2M ('singlet-to-magnetization') was applied to detect the signal. A T_{00} filter was also used in the pulse sequence to eliminate any possible magnetization that may have accumulated over the long storage times.

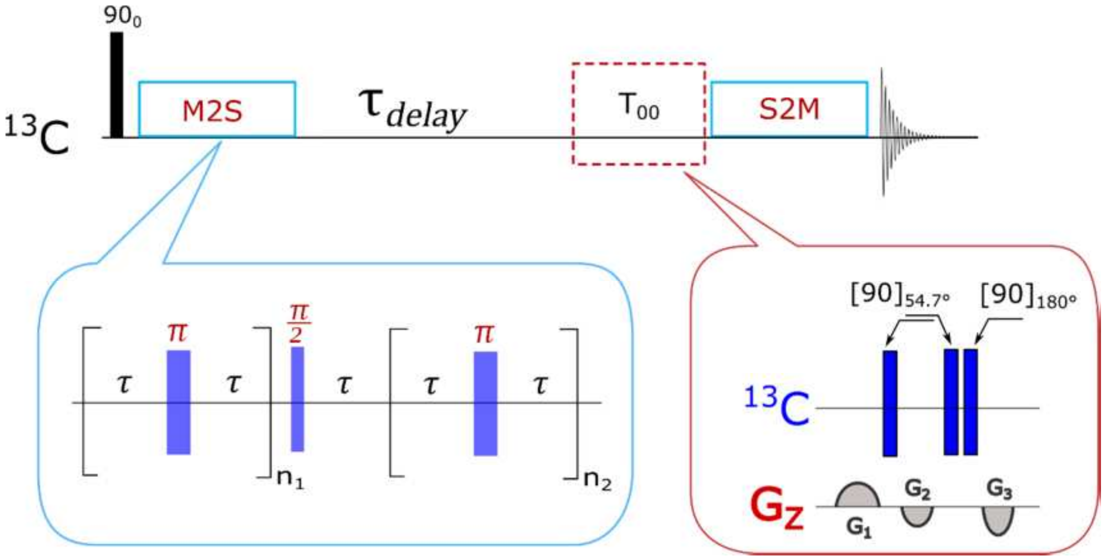

Figure S9: Pulse sequence to study long-lived singlet states.

In order to efficiently run the LLS pulse sequence, it is required to evaluate the exact parameters τ, n_1 and n_2 of Figure S9. A series of J-synchronized experiments along with Spindynamica simulations were employed to calculate those values accurately in each sample.

J-synchronization experiment

The pulse sequence for J-synchronization experiment is shown Figure S10. It consists with a series of 180˚ pulses at equal intervals. By varying the delay (τ) in between those pulses or by varying the number of 180˚ pulses (n_1), we can accurately calculate the optimized parameters required for M2S-S2M pulse sequence.

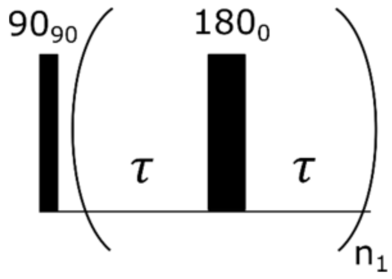

Figure S10: Pulse sequence for J-synchronized pulse sequence.

For chemically equivalent but magnetically inequivalent spin pair (*Type-1*), the theoretical values can be estimated using the following formulas,

$$\tau = {}^{1}\!/\!\left(2\sqrt{(J_{CC}+J_{HH})^2 + (\Delta J_{CH})^2}\right)$$

$$n_1 = {}^{\pi}\!/\!\left(2\tan^{-1}[\Delta J_{CH}/(J_{CC}+J_{HH})]\right)$$

Where, ΔJ_{CH} is the difference between two out of pair heteronuclear J-couplings in the spin system **1**.

For *Type-2* spin system, the theoretically estimated values are calculated as follows,

$$\tau = {}^{1}\!/\!\left(4\sqrt{J^2 + \Delta\delta^2\nu^2}\right)$$
$$n_1 = {}^{\pi}\!/\!\left(2\tan^{-1}[\Delta\delta.\nu/J]\right);$$

Where $\Delta\delta$, ν and J represent chemical shift difference, ^{13}C Larmor frequency and scalar coupling constant between the ^{13}C spin pair respectively.

In all cases, Spindynamica simulation[10] closely matches the experimental findings as shown below.

(i) Substrate-**1**: Results from varying τ, while keeping number of 180° pulses (n_1) constant.

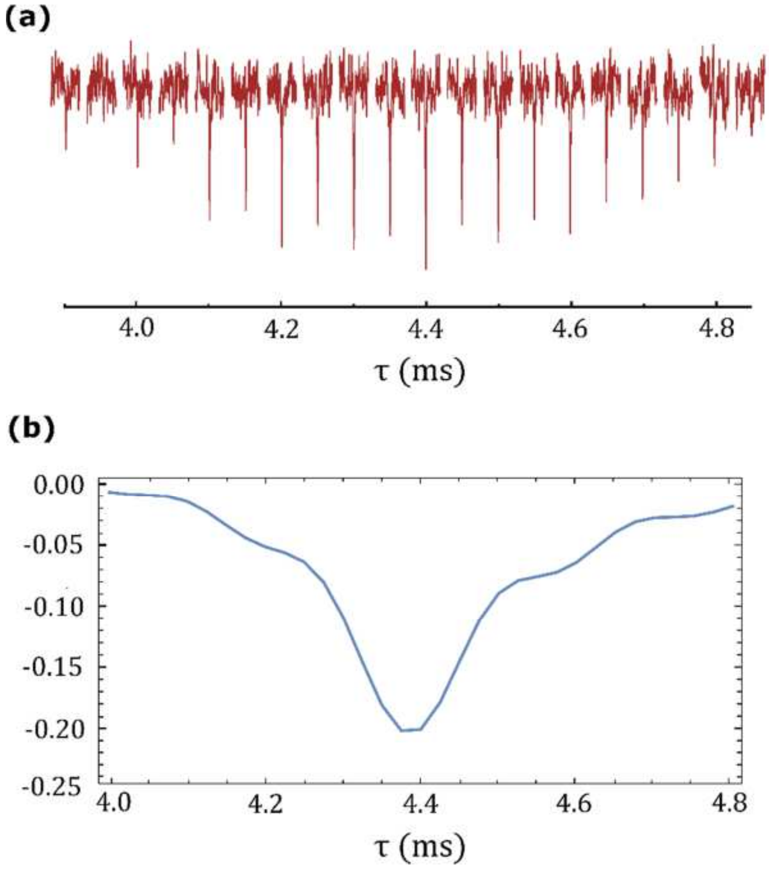

*Figure S11: (a) ^{13}C NMR spectra obtained for **1** by varying the delay parameter (τ) in J-synchronization experiments and (b) related Spindynamica simulations. An optimum efficiency is obtained when τ= 4.4 ms.*

(ii) Substrate **1**: Results from varying number of 180˚ pulses (n_1) while keeping delay ($\tau = 4.4\ \mathrm{ms}$) as constant.

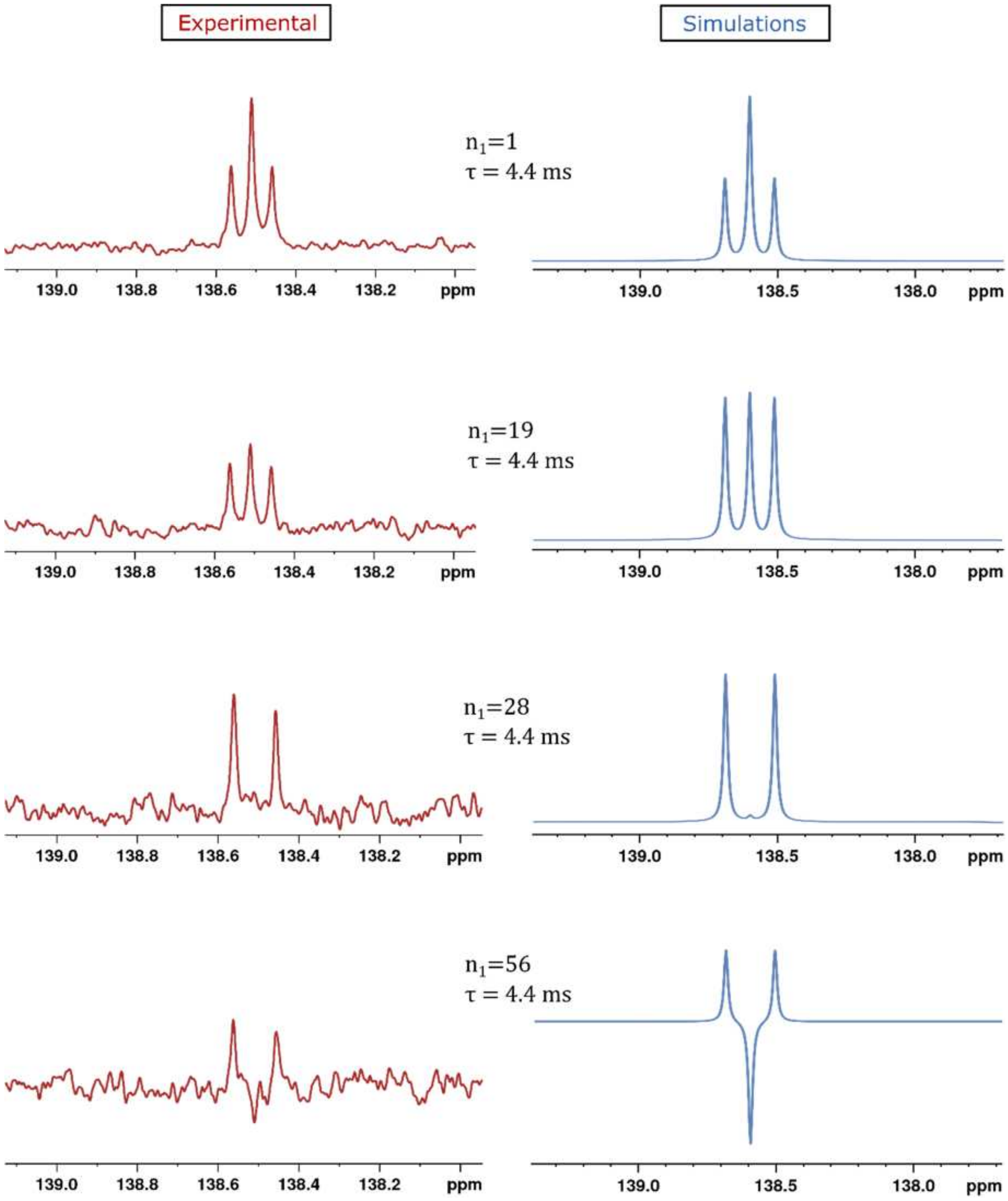

Figure S12: Left column showing experimental ^{13}C NMR spectra achieved via J-synchronized experiments with respective parameters while closely matching simulated results showing in the right column.

(iii) Substrate-**5**: Results from varying τ, while keeping number of 180˚ pulses (n_1) constant.

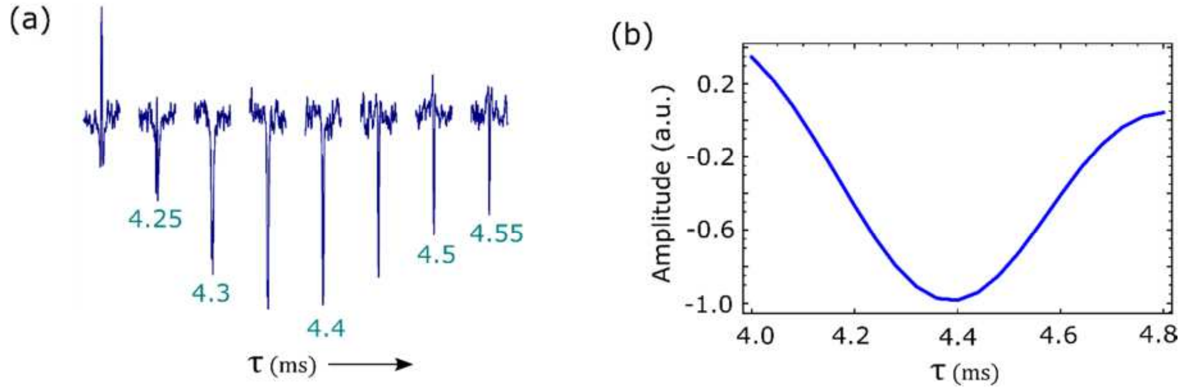

*Figure S13: (a) ^{13}C NMR spectra obtained for **5** by varying the delay parameter (τ) in J-synchronization experiments and (b) related Spindynamica simulations. An optimum efficiency is obtained when $\tau \approx 4.35$ ms.*

(iv) Substrate **5**: Results from varying number of 180˚ pulses (n_1) while keeping delay ($\tau = 4.35$ ms) as constant.

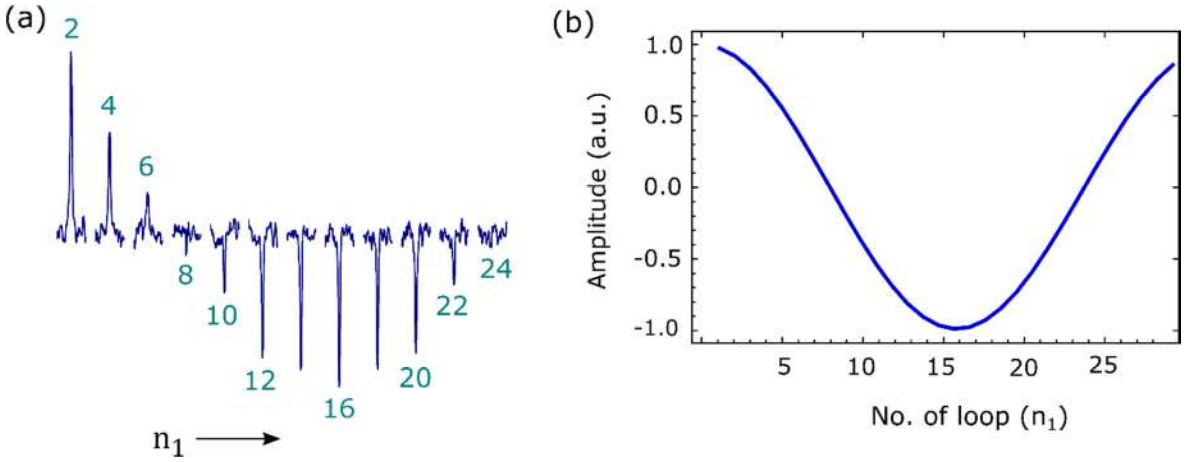

*Figure S14: (a) ^{13}C NMR spectra obtained for **5** by varying the no. of 180˚ pulse loop (n_1) in J-synchronization experiments while keeping the delay (τ) constant at 4.35 ms. (b) Corresponding Spindynamica simulations.*

Agents **4**-**7** returned similar numbers because of their comparable physical parameters i.e. chemical shifts and J-coupling values. The optimized parameters for the substrates are summarized in Table S2. Singlet lifetime constants (T_{LLS}) were measured by employing the complete sequence of M2S-S2M and lead to the results shown in Table S3. It should be noted that for agent **2**, it was not possible to extract any information by running J-synchronization due to the extremely large number of refocusing 180˚ pulses required that resulted in a very long duration pulse sequence and consequently no signal. Also fast relaxation of deuterium plays a part. In the case of agent **3**, no symmetry breaking mechanism is available due to the spin-pair's magnetically symmetric environment and hence no singlets can be populated by the means of rf pulse sequencing. Agent **8** represents a weakly coupled (J $\approx \Delta\nu$) scenario at 9.4 T and consequently low singlet lifetime.

Table S2: Experimental parameters used in the M2S-S2M pulse sequence to measure T_{LLS} for the substrates. In the case of 8, an earlier pulse sequence was used to study the T_{LLS}. † Parameters as per reference [11].

Substrate	τ (ms)	n_1	n_2
**1**	4.40	28	14
**2**	-	-	-
**3**	-	-	-
**4**	4.35	8	4
**5**	4.40	8	4
**6**	4.30	7	3
**7**	4.30	20	10
**8†**	τ_1 = 4.4 ms, τ_2 = 10.7 ms, τ_3 = 6.3 ms, τ_5 = 3.2 ms,		

Table S3. SABRE signal enhancements and lifetimes of substrates measured in high (9.4 T) and low field (~10 mT). ($\Delta\nu$ = Chemical shift difference between ^{13}C spin-pair (●) at 9.4 T in Hz; HF = high field and LF = low field). The *J*-coupling constant between two ^{13}C was found to be 58.5±2 Hz in all cases.

**Substrate No.**	**Substrate structure**	**Physical parameters @ 9.4 T**	**Enhancement (ε)/ net polarization P [%]**	**Optimum Polarization fields**	**T_1 lifetime in high field (sec)**	**Singlet Lifetime (T_S) in sec.**
**1**	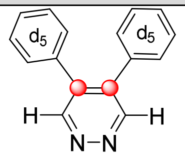	$^{2}J_{CH}$ = 6.8 Hz<br>$^{3}J_{CH}$ = 3.7 Hz<br><br>ΔJ_{CH} = 3.1 Hz	ε: 2500 ± 300<br>P ≈ 2.0 %	10 mG, 30 G, 100 G	T_1: 9.7 ± 0.3	T_S (HF): 75 ± 5.5<br>T_S (LF): 115 ± 12
**2**	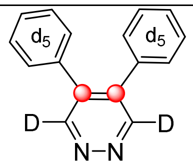	$^{2}J_{CD}$ = 1.1 Hz<br>$^{3}J_{CD}$ = 0.5 Hz<br><br>ΔJ_{CD} = 0.6 Hz	ε: 1600 ± 280<br>P ≈ 1.3 %	20 mG, 150 G	T_1: 12.4 ± 0.9	T_S (HF/LF): -
**3**	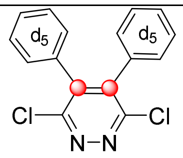	$\Delta\nu$ = 0 Hz	ε: 600 ± 50<br>P ≈ 0.5 %	20 mG	T_1: 16.0 ± 1.5	T_S (HF/LF): No access
**4**	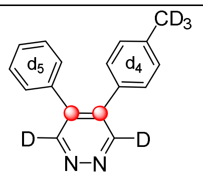	$\Delta\nu$ = 11.0 Hz	ε: 1600 ± 300<br>P ≈ 1.3 %	20 mG, 150 G	T_1: 10.2 ± 0.6	T_S (HF): 22 ± 3.0<br>T_S (LF): 28 ± 6.5
**5**	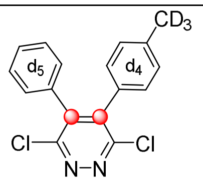	$\Delta\nu$ = 10.4 Hz	ε: 550 ± 50<br>P ≈ 0.45 %	5 mG	T_1: 15.5 ± 1.2	T_S (HF): 90 ± 3.0<br>T_S (LF): 165 ± 18
**6**	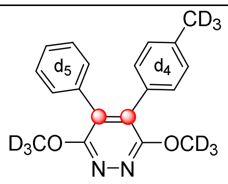	$\Delta\nu$ = 14.5 Hz	ε: 350 ± 40<br>P ≈ 0.35 %	10 mG	T_1: 10.4 ± 0.3	T_S (HF): 115 ± 5.5<br>T_S (LF): 148 ± 20
**7**	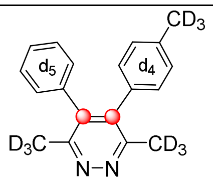	$\Delta\nu$ = 4.4 Hz	ε: 600 ± 50<br>P ≈ 0.50 %	1 mG	T_1: 15.2 ± 0.3	T_S (HF): 145 ± 6<br>T_S (LF): 186 ± 18
**8**	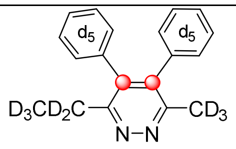	$\Delta\nu$ = 78.8 ± 0.5	ε: 800 ± 150<br>P ≈ 0.65 %	10 mG	T_1: 7.5 ± 0.5	T_S (HF) < 5<br>T_S (LF): 45 ± 6
**9**	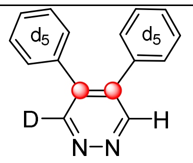	$^{2}J_{CH}$ = 6.8 Hz<br>$^{3}J_{CD}$ = 0.5 Hz<br>$^{2}J_{CD}$ = 1.1 Hz<br>$^{3}J_{CH}$ = 3.7 Hz	ε: 800 ± 200<br>P ≈ 0.65 %	30 G, 100 G	T_1: 8.7 ± 1.2	T_S (HF): < 2 s

S7. NMR spectral data

The following examples of NMR spectra reflect the substrate spin systems studied here. Samples were prepared as described in section S1. All spectra were measured in a 400 MHz NMR spectrometer at room temperature.

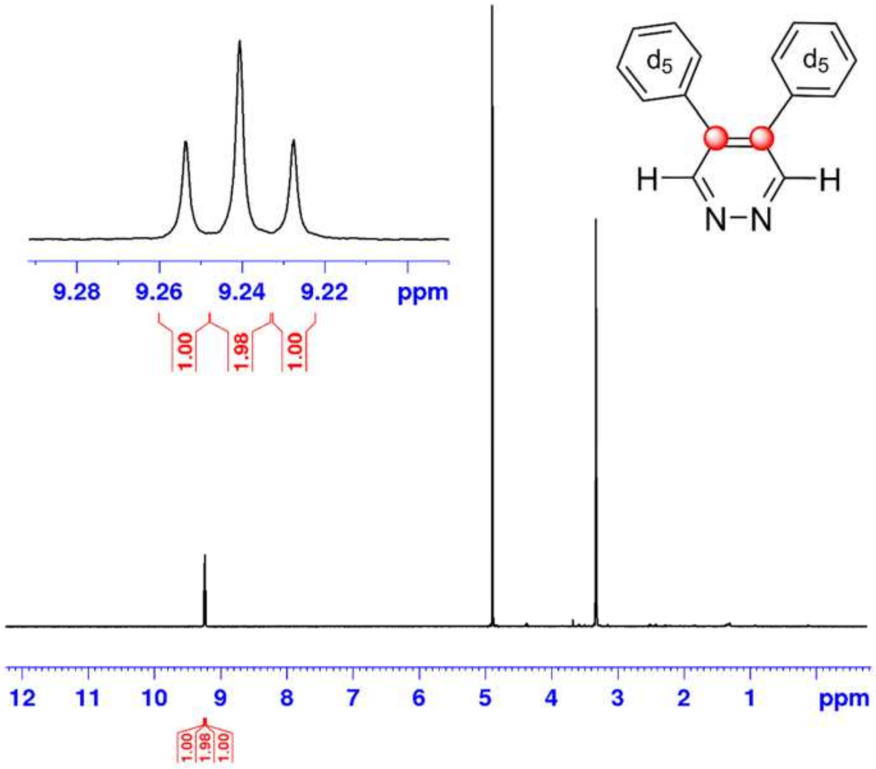

*Figure S15: ^{1}H NMR spectrum of **1** (inset) dissolved in methanol-d_4 with [IrCl(COD)(IMes)].*

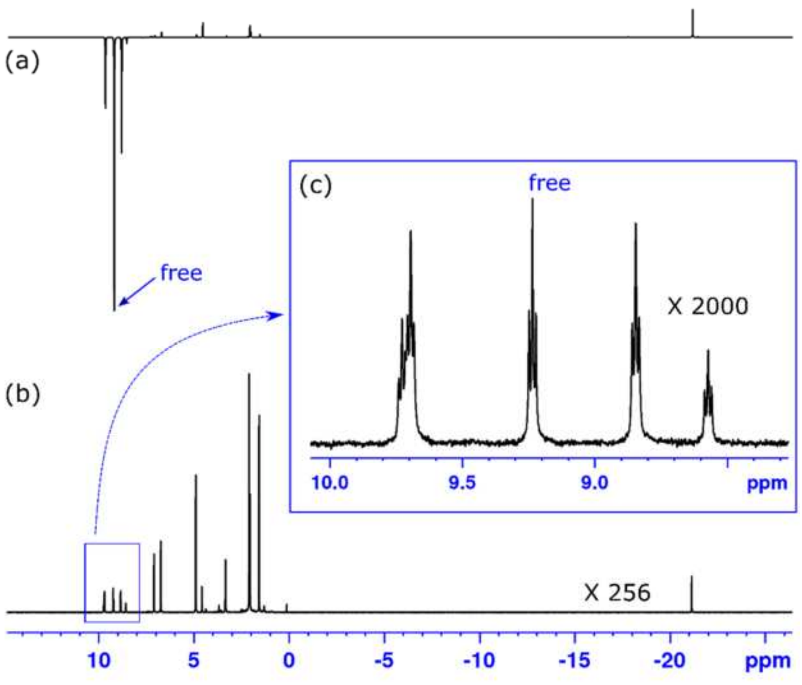

*Figure S16: ^{1}H NMR spectra of **1** dissolved in methanol-d_4 with the IMes catalyst to observe (a) ^{1}H SABRE when polarized in a 60 G mixing field and (b) ^{1}H thermal signal where vertical scale multiplied by 256 times for comparison. In the inset box (c) the substrate signal is vertically scaled to clearly show the free and bounds peaks detectable after the SABRE process.*

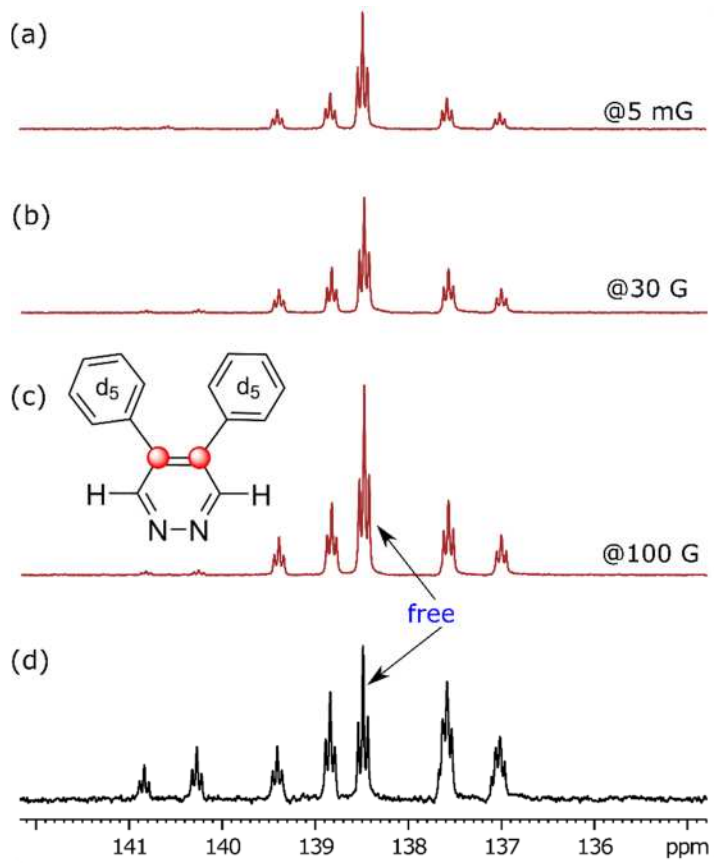

Figure S17: ^{13}*C NMR spectra of* ***1*** *when applying 90° pulse on both channels (a)* ^{13}*C SABRE at 5 mG; (b) at 30 G; (c) at 100 G; (d) thermally polarized spectrum acquired over 2000 transients.*

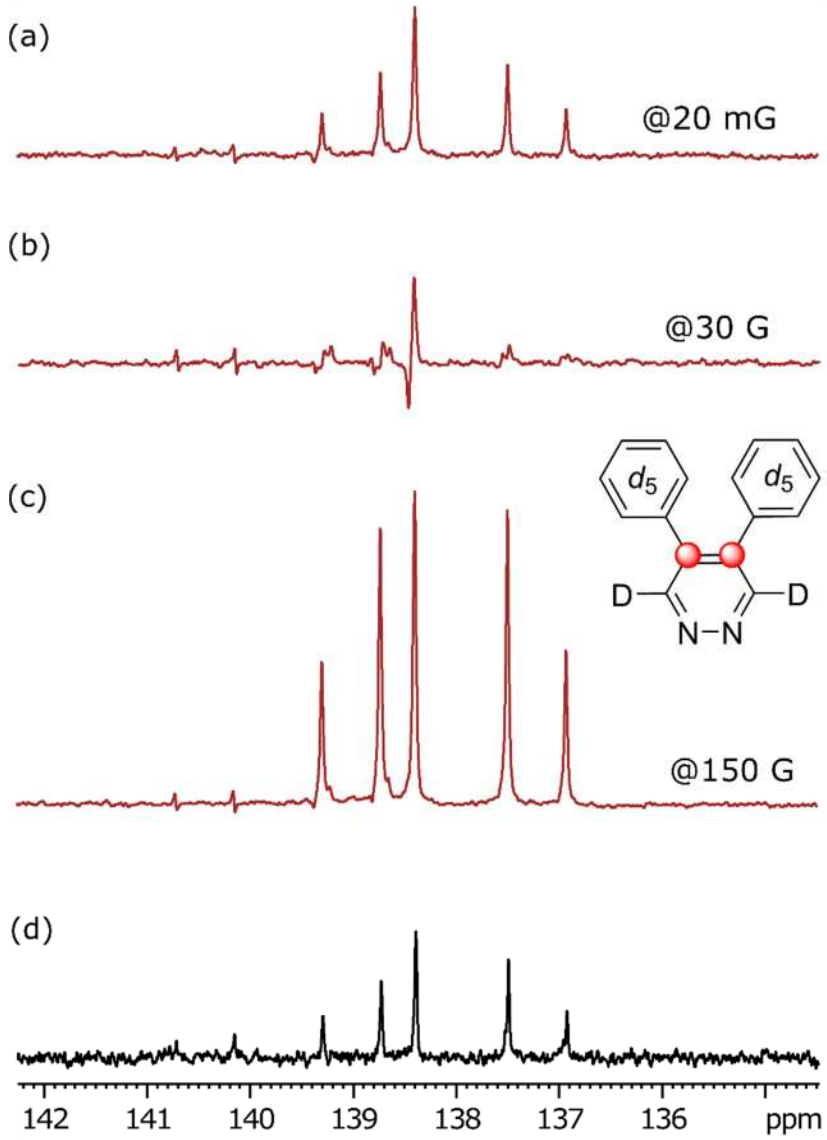

*Figure S18: ^{13}C NMR spectra of **2** dissolved in methanol-d_4 with the IMes catalyst to observe SABRE: (a) ^{13}C SABRE at 20mG; (b) at 30 G; (c) at 150 G; and (d) thermally polarized spectrum of 500 transients for comparison. The resonance frequency of ^{13}C pair appears at the center of these spectra while all other signals represent bound peaks.*

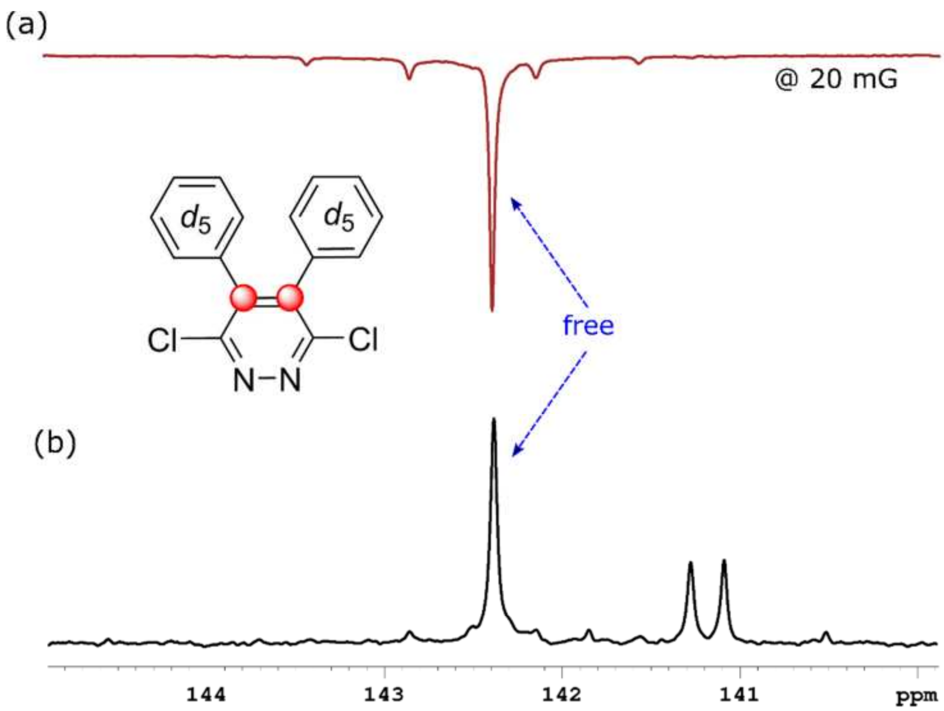

*Figure S19: ^{13}C NMR spectra of **3** dissolved in methanol-d$_4$ with the IMes catalyst to observe SABRE (a) at 20 mG mixing field and (b) the thermally equilibrated spectrum acquired over 800 transients. The resonance frequency of ^{13}C pair appears at the center of these spectra while all other signals represent bound peaks.*

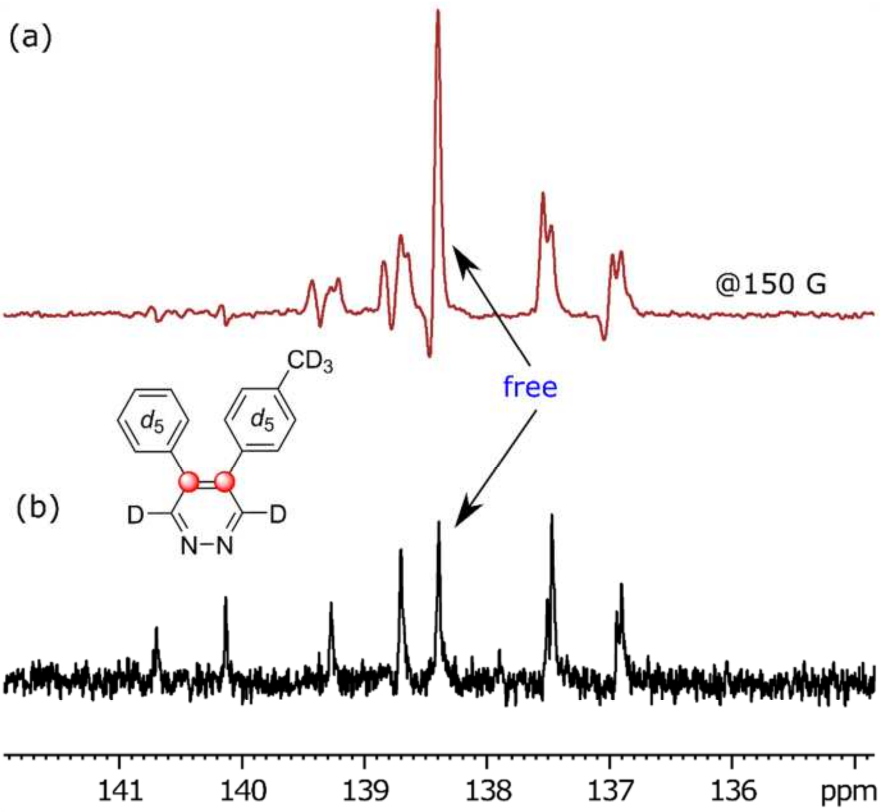

*Figure S20: (a) ^{13}C NMR spectra of **4** dissolved in methanol-d$_4$ with the IMes catalyst to observe SABRE and (b) the thermally equilibrated spectrum of 100 transients and vertically scaled by 4. The resonance frequency of the ^{13}C pair appears at the center of these spectra while all other signals represent different bound peaks.*

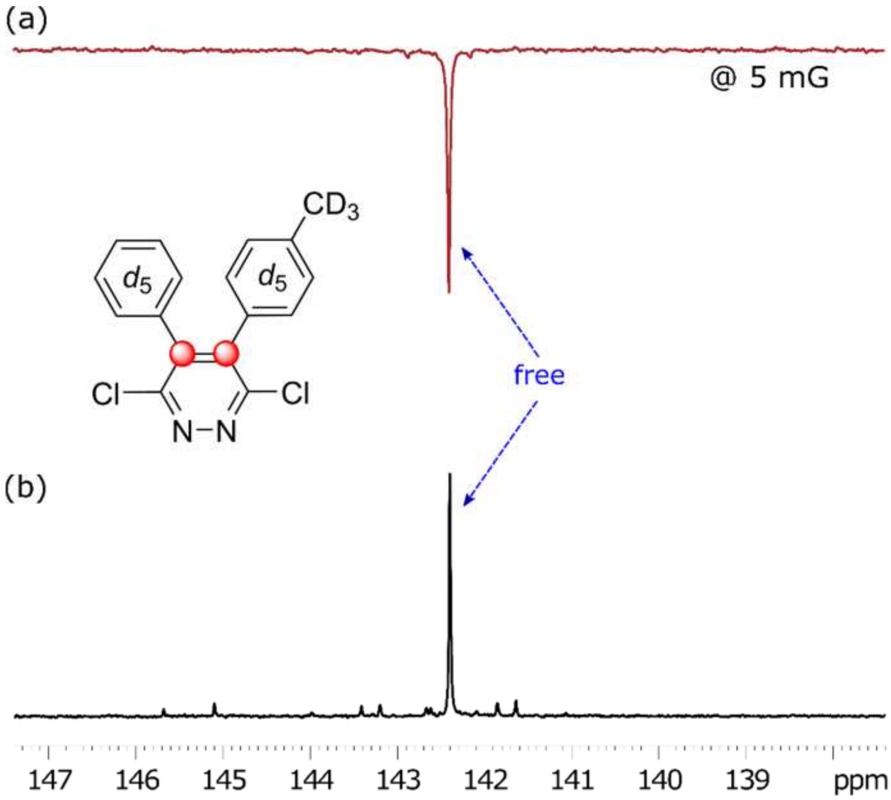

*Figure S21: ^{13}C NMR spectra of **5** dissolved in methanol-d$_4$ with the IMes catalyst to observe SABRE (a) at 5 mG mixing field and (b) thermally equilibrated spectrum of 400 transients. The 'free' resonance signals of the ^{13}C pair appear at the center of these spectra.*

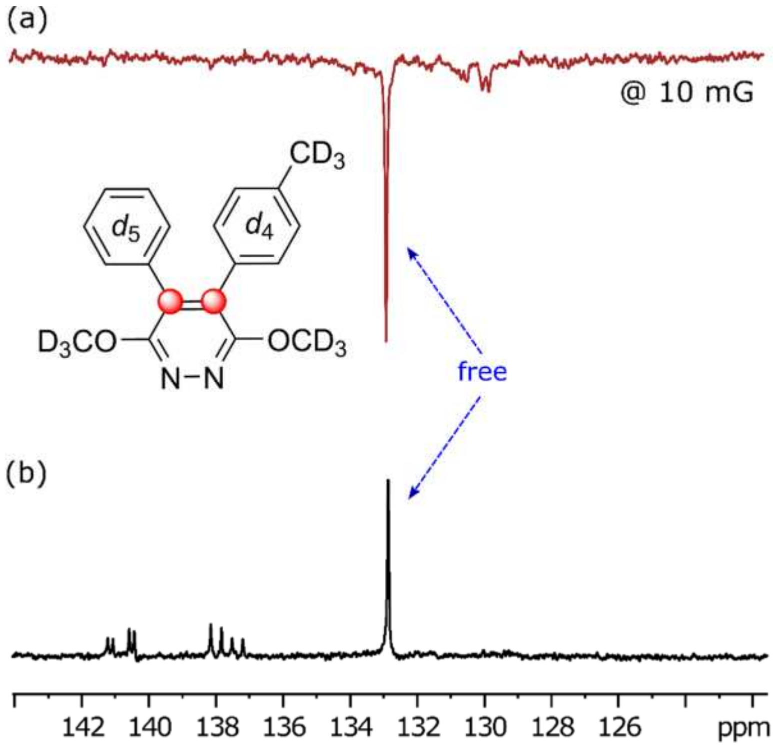

*Figure S22: ^{13}C NMR spectra of **6** dissolved in methanol-d$_4$ with the IMes catalyst to observe SABRE (a) at 10 mG mixing field and (b) thermally equilibrated spectrum acquired over 200 transients. The resonance frequency of ^{13}C pair appears at the center of these spectra while all other signals represent different bound peaks.*

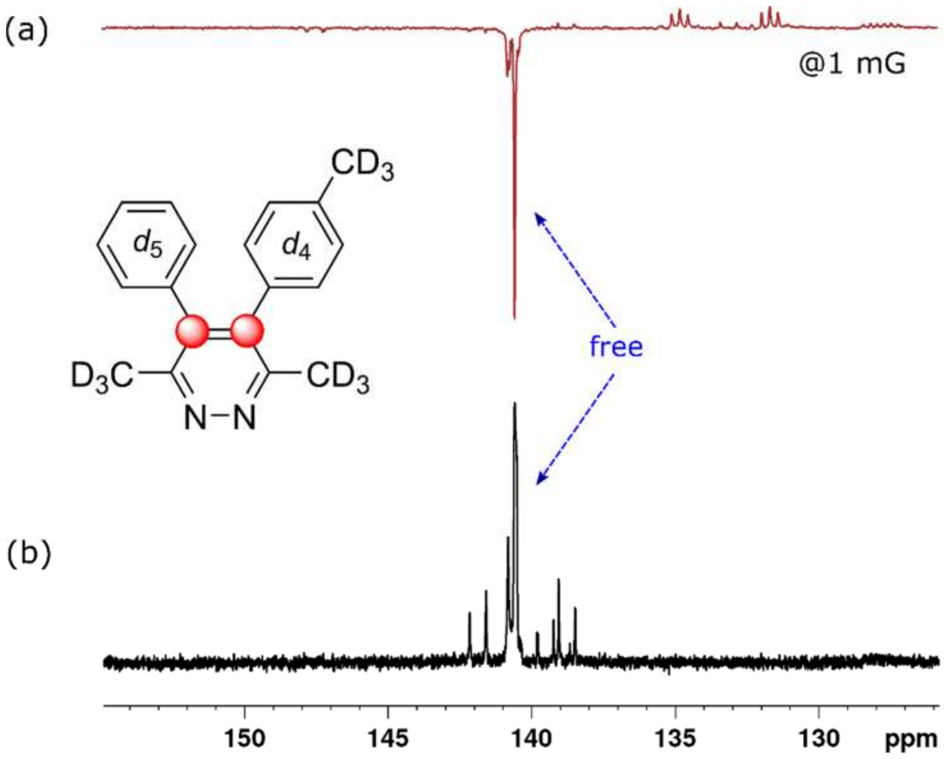

Figure S23: ^{13}C NMR spectra of **7** dissolved in methanol-d_4 with the IMes catalyst to observe SABRE (a) at 1 mG mixing field and (b) thermally equilibrated spectrum acquired over 200 transients and vertically scaled by 2. The resonance frequency of ^{13}C pair appears at the center of these spectra while all other signals represent different bound peaks.

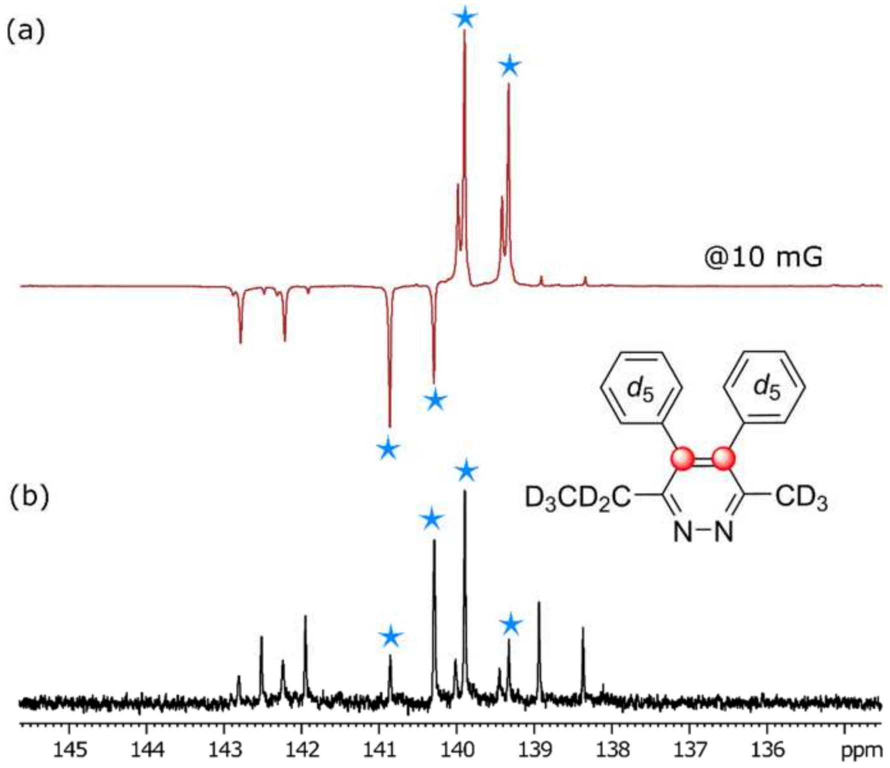

Figure S24: (a) ^{13}C NMR spectra of **8** dissolved in Methanol-d_4 with the IMes catalyst to observe SABRE and (b) the thermally equilibrated spectrum of 400 transients, further vertically scaled 2 times. The 'free' resonance signals of the ^{13}C pair appear at the center of these spectra and are denoted by ⋆; while all other signals represent different 'bound' peaks.

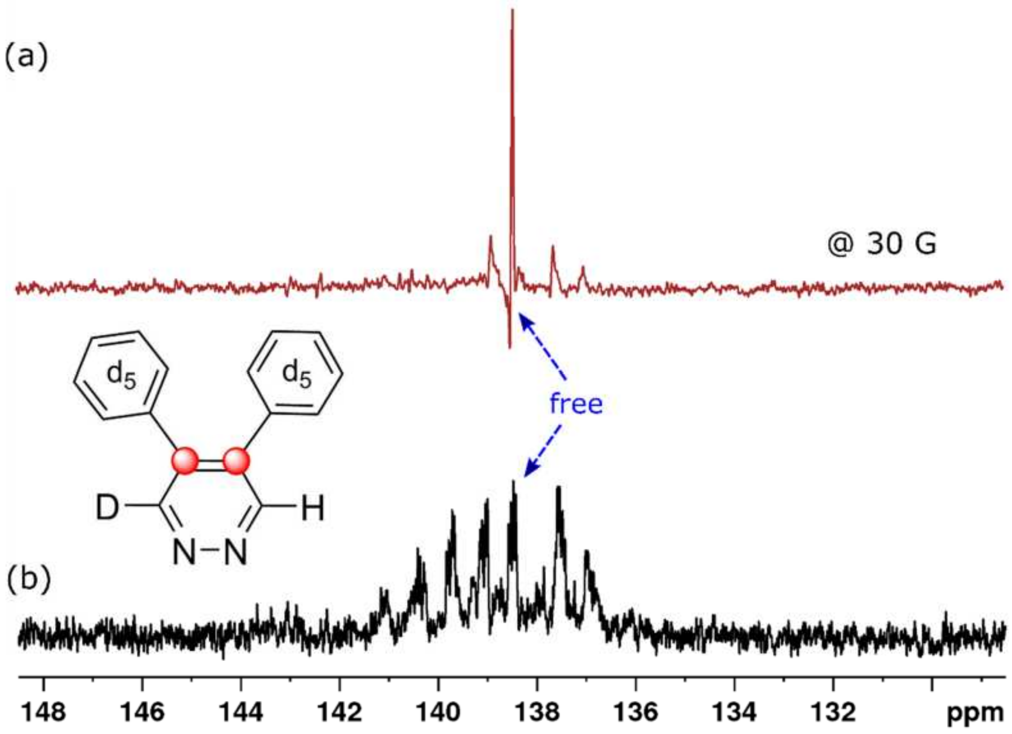

*Figure S25: ^{13}C NMR spectra of **9** dissolved in methanol-d_4 with the IMes catalyst to observe SABRE (a) at 30 G mixing field and (b) thermally equilibrated spectrum of 100 transients which vertically scaled by 4. The 'free' resonance signals of the ^{13}C pair appear at the center of these spectra.*

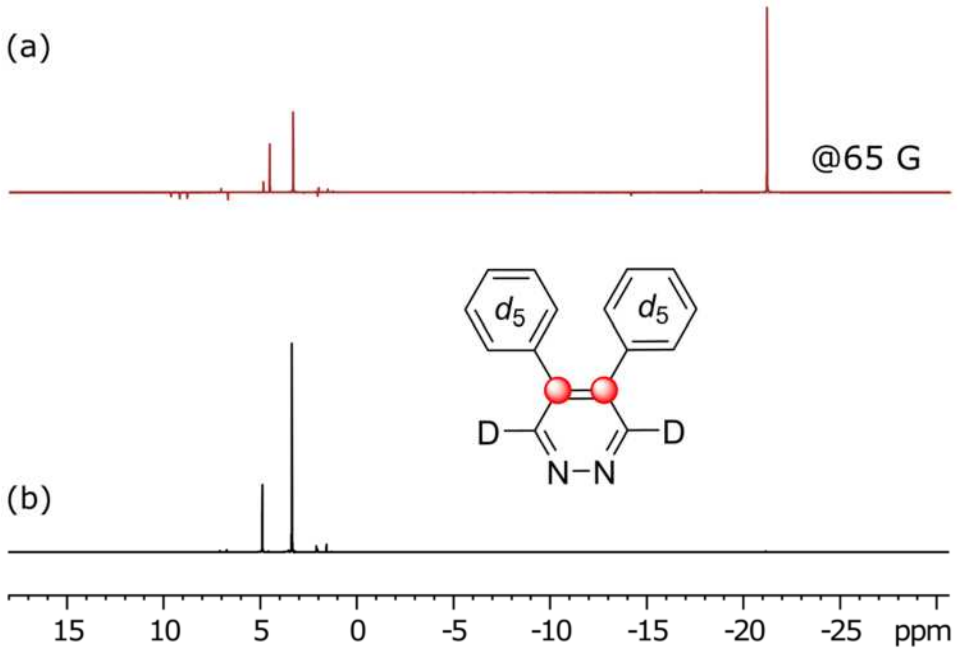

*Figure S26: (a) ^{1}H NMR spectrum of agent **2** dissolved in methanol-d_4 with the IMes catalyst showing SABRE transfer at 65 G showing minimal transfer in the 1H NMR spectrum in accordance with the high level of ^{2}H labelling. (b) analogous thermally polarized ^{1}H NMR trace.*

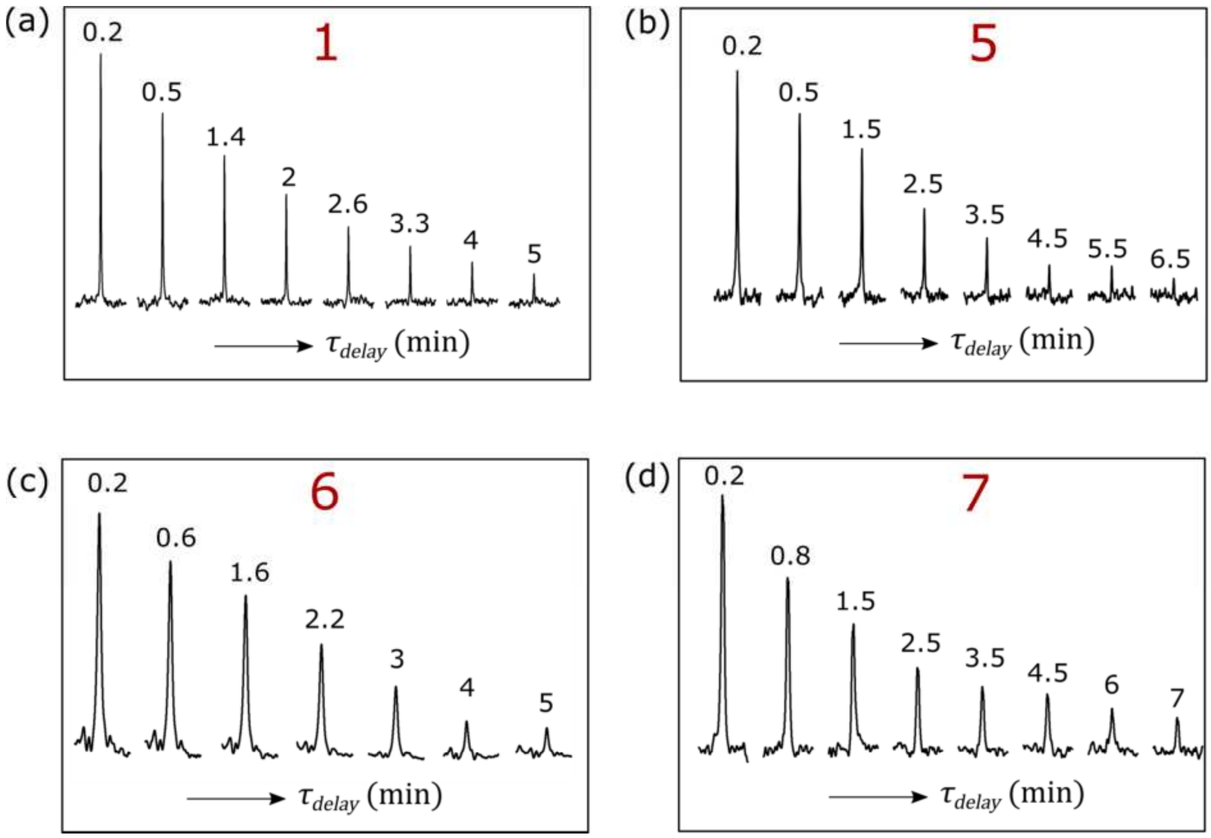

Figure S27: Series of ^{13}C-NMR responses as a function of delay after singlet state creation (mins) showing the decay of the hyperpolarized ^{13}C response for agents 1 (a), 5 (b), 6 (c) and 7 (d). These traces correspond to those that were used in producing Fig. 3.

S8. References

[1] K. D. Atkinson, M. J. Cowley, P. I. P. Elliott, S. B. Duckett, G. G. R. Green, J. Lopez-Serrano, A. C. Whitwood, *J. Am. Chem. Soc.* **2009**, *131*, 13362-13368.

[2] R. E. Mewis, et al., *Magn. Res. Chem.* **2014**, *52*, 358-369.

[3] M. Carravetta, O. G. Johannessen, M. H. Levitt, *Phys. Rev. Lett.* **2004**, *92*, 153003.

[4] L. D. Vazquez-Serrano, B. T. Owens, J. M. Buriak, *Inorganica Chimica Acta* **2006**, *359*, 2786-2797.

[5] K. M. Appleby, R. E. Mewis, A. M. Olaru, G. G. R. Green, I. J. S. Fairlamb, S. B. Duckett, *Chemical Science* **2015**, *6*, 3981-3993.

[6] S. S. Roy, P. Norcott, P. J. Rayner, G. G. R. Green, S. B. Duckett, *Angew. Chem. Int. Ed.* **2016**, *55*, 15642-15645.

[7] S. S. Roy, P. J. Rayner, P. Norcott, G. G. R. Green, S. B. Duckett, *Phys. Chem. Chem. Phys.* **2016**, *18*, 24905-24911.

[8] R. A. Green, R. W. Adams, S. B. Duckett, R. E. Mewis, D. C. Williamson, G. G. R. Green, *Prog. Nucl. Mag. Res. Sp.* **2012**, *67*, 1-48.

[9] T. Theis, M. L. Truong, A. M. Coffey, R. V. Shchepin, K. W. Waddell, F. Shi, B. M. Goodson, W. S. Warren, E. Y. Chekmenev, *J. Am. Chem. Soc.* **2015**, *137*, 1404-1407.

[10] SpinDynamica, Code for Mathematica, Programmed by Malcolm H. Levitt, with Contributions by Jyrki Rantaharju, Andreas Brinkmann, and Soumya Singha Roy., http://www.spindynamica.soton.ac.uk/)..

[11] M. Carravetta, M. H. Levitt, *J. Am. Chem. Soc.* **2004**, *126*, 6228-6229.
